# Food-derived linear vs. rationally designed cyclic peptides as potent TNF-alpha inhibitors: an integrative computational study

**DOI:** 10.3389/fbinf.2025.1716375

**Published:** 2025-11-18

**Authors:** Manisha Shah, Sivakumar Arumugam

**Affiliations:** Department of Bio-Sciences, School of Bio Sciences and Technology, Vellore Institute of Technology, Vellore, Tamil Nadu, India

**Keywords:** food-derived peptide, TNF-alpha, interface residue, molecular docking, cyclic peptide, linear peptide, disulfide-bridge peptide

## Abstract

**Introduction:**

Tumor necrosis factor-alpha (TNF-alpha) is a central mediator of chronic inflammation and a validated therapeutic target in atherosclerosis and related cardiovascular disorders. Peptide therapeutics offer high specificity and low toxicity; however, few natural sequences have been optimized for durable TNF-alpha inhibition.

**Methods:**

A dual in silico strategy was employed to identify potent inhibitors: (i) virtual screening of experimentally validated food-derived bioactive peptides and (ii) rational design of an N-C cyclized and disulfide-bridge peptide based on the TNF-alpha-TNFR1 interface. Molecular docking, 200-ns molecular dynamics simulations, and MM/PBSA free-energy analyses were performed.

**Results:**

The selected peptides exhibited strong and persistent interactions with key TNF-alpha residues, particularly Tyr119. The cyclic analogue demonstrated deeper free-energy minima, higher binding affinity, and more stable hydrogen-bond networks than the linear sequence. ADMET profiling revealed superior metabolic stability, reduced plasma clearance, and no predicted cardiotoxicity.

**Discussion:**

These results indicate that dietary peptides can serve as templates for TNF-alpha inhibition, and interface-guided cyclization rationally enhances stability, binding affinity, and drug-like properties. This study provides a mechanistic framework for developing food-derived peptides as next-generation TNF-alpha antagonists and supports United Nations SDGs 3 and 9 by promoting innovative, low-toxicity therapeutics for chronic inflammation and cardiovascular diseases.

## 1 Introduction

Atherosclerosis persists as a major global cause of death and disability (Bonow et al., 2002), primarily driven by chronic, low-grade inflammation that disrupts vascular equilibrium. Among the mediators sustaining this pro-inflammatory state, tumor necrosis factor-alpha (TNF-alpha) plays a pivotal role (Kalliolias and Ivashkiv, 2016; Muller et al., 2018). Originally identified as “cachectin” (Beutler and Cerami, 1986), TNF-alpha was later recognized as a key mediator of endothelial dysfunction and plaque progression in atherosclerosis (Feingold et al., 1998; Grunfeld and Feingold, 1991). TNF-alpha is produced as a 233-amino-acid transmembrane precursor cleaved by TNF-alpha converting enzyme to release the active soluble form (Itai et al., 2001; Pennica et al., 1984), which signals mainly through TNFR1 (pro-inflammatory) and TNFR2 (regenerative) (Ma et al., 2015). Mapping the precise TNF-alpha/TNFR1 interface residues provides a structural framework for developing short inhibitory peptides capable of competitively blocking this interaction.

Therapeutic neutralization of TNF-alpha using monoclonal antibodies such as infliximab has proven effective in autoimmune and inflammatory disorders (Jang et al., 2021). However, the large molecular size and foreign immunogenic epitopes of biologics contribute to adverse effects, including anaphylaxis, serum sickness, and cytokine release syndrome. Reports have also linked infliximab to hepatotoxicity and thrombocytopenia (Dang et al., 2014; Matsumoto and Mashima, 2016). Moreover, clinical reviews indicate that while TNF-alpha blockage improves subclinical vascular inflammation, its cardiovascular outcomes can vary depending on the diseases context. These limitations underscore the demand for alternative, selective inhibitors with improved safety and pharmacokinetics profiles.

**FIGURE 1:**
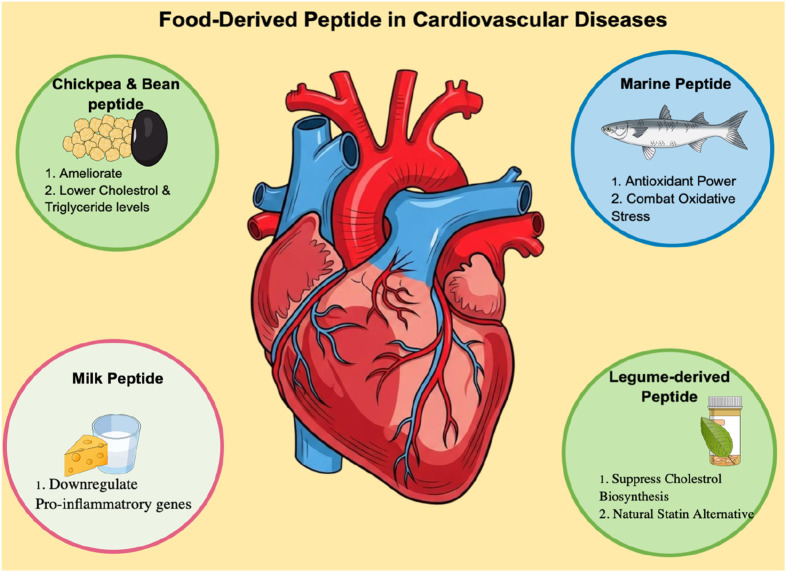
Role of food-derived peptides in cardiovascular health.

Therapeutic peptides offer a promising approach to fill this gap. The smaller molecular size, higher target specificity, and intrinsic biodegradability result in reduced toxicity and minimal immunogenicity compared to antibody-based biologics. Rationally designed peptides can mimic receptor-binding epitopes to achieve high-affinity TNF-alpha inhibition with lower systemic risk, greater tissue permeability, and controlled clearance (Wang et al., 2023). In parallel, food protein-derived peptides have emerged as natural, multifunctional agents that regulate lipid metabolism, oxidative stress, and inflammatory signaling in both *in vitro* and *in vivo* (Majumder et al., 2016). For instance, chickpea peptides produced through alcalase hydrolysis alleviate dyslipidaemia by reducing plasma and hepatic lipid levels in high-fat diet models (Shi et al., 2019), while bean hydrolysates exhibit hypocholesterolemic effects in BALB/c mice (Gomes et al., 2020). Marine collagen-derived peptides have shown antioxidant potential and functional applicability in geriatric nutrition (Kumar A. et al., 2019), Likewise, milk-derived tripeptides IPP and VPP significantly reduced atherosclerotic lesion size in ApoE knockout mice by downregulating TNF-alpha and other pro-inflammatory genes (Nakamura et al., 2013). While statins remain the clinical mainstay for managing dyslipidemia *via* 3-hydroxy-3-methylglutaryl-CoA reductase inhibition, their adverse muscle and hepatic effects limit long-term use. In contrast, legume-derived peptides inhibit cholesterol biosynthesis with excellent safety margins (Fonseca Hernandez et al., 2024; Kumar V. et al., 2019). Recent findings emphasize that certain food-derived peptides also downregulate TNF-alpha–NF-κB signaling and promote endothelial protection, revealing their dual nutraceutical and therapeutic value. These cumulative insights establish a strong foundation for exploring peptide-based TNF-alpha modulators that combine the biochemical efficacy of biologics with the safety advantages of natural bioactives (Kotlyarov, 2025). An overview of key food-derived peptides and their reported cardioprotective mechanisms is shown in Figure 1.

**FIGURE 2:**
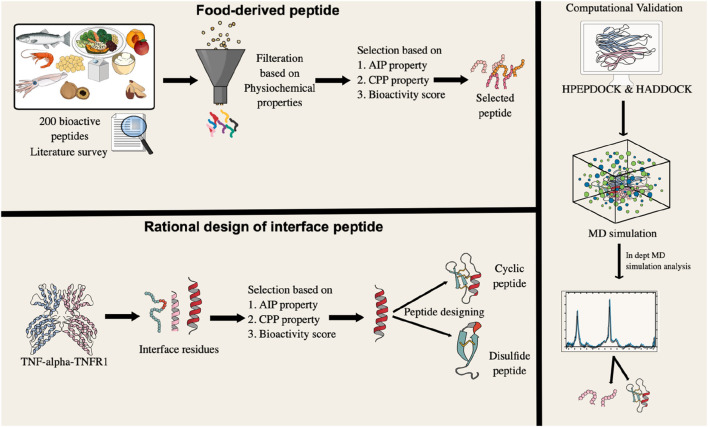
A graphical representation of the research methodology, outlining the sequential steps undertaken from data acquisition to result interpretation.

Despite these findings, few dietary peptides have been computationally evaluated for direct TNF-alpha inhibition. To explore this potential, we assembled a library of 200 experimentally validated bioactive peptides derived from diverse dietary sources, including milk, marine organisms, vegetables, fruits, and legumes such as lentils, beans, *etc.* Although many of these peptides show anti-inflammatory, antihypertensive, and antioxidant effects, their therapeutic promise has rarely been examined computationally. To address this gap, we adopted a two-step strategy. First, we performed virtual screening of the peptide library against TNF-alpha to identify high-affinity binders. Second, rational design of N-C cyclic and disulfide-bridge peptides based on the TNF-alpha–TNFR1 interface. Our approach aimed to not only identify high-affinity binders but also to critically evaluate whether structural modifications truly enhance conformational rigidity and binding stability, a common assumption in the literature. This integrative study provides a mechanistic framework for advancing food-derived peptides as next-generation TNF-alpha antagonists. By leveraging naturally sourced bioactive peptides and computational design, this work aligns with the United Nations Sustainable Development Goals particularly SDG 3 (Good Health and Well-Being) and SDG 9 (Industry, Innovation, and Infrastructure) by promoting sustainable, innovation-driven approaches for cardiovascular drug discovery. The overall methodology is presented in Figure 2.

## 2 Materials and methods

### 2.1 Literature survey and peptide collection

A systematic literature review was performed to identify experimentally validated bioactive peptides derived from natural dietary sources, including milk, marine organisms, lentils, vegetables, and fruits. A total of 200 peptides were collected from peer-reviewed studies reporting anti-inflammatory, antihypertensive, and antioxidant activities supported by *in vitro* and *in vivo* evidence. Peptide sequences, source organisms, and biological activities were manually curated to construct the peptide dataset for subsequent *in silico* screening.

### 2.2 Peptide selection and classification

The physicochemical properties of the selected peptides were evaluated using PepCalc (https://pepcalc.com/) and ExPASy ProtParam (https://web.expasy.org/protparam/) to obtain parameters including molecular weight, theoretical isoelectric point (pI), amino acid composition, number of positively and negatively charged residues, net charge, extinction coefficient, estimated half-life, net charge, instability index, and grand average of hydropathicity (GRAVY). Toxicity prediction was performed using ToxIBTL (https://server.wei-group.net/ToxIBTL/Server.html), a deep learning-based classifier for toxic *versus* non-toxic peptides. Only non-toxic and physicochemically stable peptides were retained for further analyses (Wei et al., 2022).

### 2.3 Systematic evaluation of peptide functional properties

The shortlisted peptides were evaluated for their potential bioactivity, cell-penetrating ability, peptide bioactivity score, and anti-inflammatory properties using multiple computational predictors. General bioactivity scores were calculated with PeptideRanker (http://distilldeep.ucd.ie/PeptideRanker), where peptides scoring above 0.5 were considered biologically active. The CellPPD server (https://webs.iiitd.edu.in/raghava/cellppd), was employed to assess cell-penetrating potential through an SVM-based model trained on experimentally validated CPPs (Gautam et al., 2015). Anti-inflammatory activity was further predicted using AIPpred (http://www.thegleelab.org/AIPpred/) (Manavalan et al., 2018) which integrates amino acid and dipeptide composition-based features within a random forest framework. Peptides that demonstrated consistently high scores across these platforms were prioritized for modeling.

### 2.4 Three-dimensional structural prediction of food-derived peptide

The three-dimensional conformations of the selected peptide molecules were predicted using the PEP-FOLD4.0 web server (http://bioserv.rpbs.univ-paris-diderot.fr/services/PEP-FOLD4) (Rey et al., 2023). PEP-FOLD4 is a robust *de novo* prediction tool that generates optimized, low-energy peptide structures. The resulting models were used for subsequent molecular docking and stability analyses. Detailed descriptions of the PEP-FOLD4 fragment-based modeling approach and sOPEP force field parameters are provided in the Supplementary Material (Section 1.1).

### 2.5 Identification and rational design of interacting peptides

The crystal structure of the TNF-alpha–TNFR1 complex (PDB ID: 7KPB) (Lightwood et al., 2021) was retrieved from the Protein Data Bank and analyzed in PyMOL (version 3.1.5.1) to identify protein–protein interacting residues. Three distinct TNFR1 regions involved in TNF-alpha binding were identified, and the longest interacting peptide region was selected for further study due to its potential for stable interactions. To enhance stability and functional properties, rational redesign was performed by generating cyclic and disulfide-stabilized analogues. Cyclic peptides were modeled *via* N-to-C terminal covalent cyclization to improve conformational rigidity and proteolytic resistance, while disulfide-stabilized peptides were designed by introducing cysteine residues to form S–S bridges, reinforcing structural robustness. All peptide variants were modeled using the PEPstrMOD server (Singh et al., 2015), which incorporates secondary structure prediction, torsion angle constraints, and energy minimization. The resulting three-dimensional structures were used for downstream docking and molecular dynamics analyses.

### 2.6 Docking protocol

Molecular docking was performed to investigate interactions of TNF-alpha with both food-derived and rationally designed peptides. The crystal structure of TNF-alpha (PDB ID: 2AZ5) (He et al., 2005) was retrieved from the Protein Data Bank and prepared by removing water molecules and heteroatoms, followed by addition of hydrogens and charges using AutoDock tools (v1.5.6). The co-crystallized ligand was extracted and used as a reference. Initial docking was conducted using HPEPDOCK 2.0, allowing flexible and cyclic peptide docking, with experimentally validated TNF-alpha active site residues (A:57, A:59–61, A:119–122, and A:151) defined as the binding interface. The ten top-ranked docking poses were shortlisted for further analysis (Zhou et al., 2018). To validate and complement these results, docking was further performed using the HADDOCK 2.4 (High Ambiguity Driven protein–protein DOCKing) server, an information-driven flexible docking platform. Docking restraints were applied to the validated active-site residues, and resulting complexes were evaluated using the HADDOCK score, calculated as:

$$\text{HADDOCK score}=1.0E_{\text{vdW}}+0.2E_{\text{elec}}+1.0E_{\text{desolv}}+0.1E_{\text{air}}$$

Where E_vdW_ is the van der Waals energy, E_elec_ is the electrostatic energy, E_desolv_ is the desolvation energy, and E_air_ is the restraint violation energy. Lower HADDOCK scores indicate more favorable and stable complexes. Docked complexes were visualized and analyzed in PyMOL (version 3.1.5.1) to identify key hydrogen-bonding, hydrophobic, and electrostatic interactions at the protein–peptide interface (Honorato et al., 2024).

### 2.7 Molecular dynamics simulation

Molecular dynamics (MD) simulations were performed for both complexes, with system preparation carried out *via* the CHARMM-GUI Solution Builder (Lee et al., 2019). Each complex was immersed in a TIP3P water box with a 15 Å buffer in all spatial dimensions. To mimic physiological ionic conditions and achieve charge neutrality, 0.15 M sodium chloride (NaCl) was added to the solvent, and the overall system charge was balanced. The relaxation phase included two stages of energy minimization followed by a single equilibration step. During equilibration, the system’s temperature was controlled at 303.15 K using the Nose–Hoover thermostat, while the pressure was maintained at 1.0 bar *via* semi-isotropic Parrinello–Rahman pressure coupling. The LINCS algorithm was used to constrain bonds, and non-bonded interactions were treated using the Verlet cutoff method. The production run was executed for 200 nanoseconds using a 2-femtosecond time step, accumulating approximately 100 million integration steps. Ligand topology and parameters were generated using the Ligand Reader and Modeler module of CHARMM-GUI, which applies the CHARMM General Force Field (CGenFF) to produce force field-compatible input files (Kim et al., 2017). All simulations were conducted with GROMACS version 2021.4. The resulting trajectories were carefully analyzed to assess the structural stability of the complexes through calculations of RMSD, RMSF, Rg, and hydrogen bond interactions.

### 2.8 Advanced trajectory analysis

To analyze the molecular dynamics trajectories of the protein–peptide complexes, a multi-layered approach was employed, integrating Principal Component Analysis (PCA), secondary structure evolution *via* the Dictionary of Secondary Structure of Proteins (DSSP), and Free Energy Landscape (FEL) mapping. Structural interpretation was performed using the Geo_measure 0.9 plugin in PyMOL (version 3.1.5.1) (Kagami et al., 2020). FELs were constructed from Root Mean Square Deviation (RMSD) and Radius of Gyration (Rg) values, where RMSD measured deviations relative to the reference conformation, and Rg provided insights into overall compactness. FEL analysis identified thermodynamically favorable conformational states and transitional intermediates, providing a detailed view of conformational stability and dynamic fluctuations.

Secondary structure evolution was monitored with DSSP, classifying residues as helices (H), β-strands (E), or coils (C). DSSP profiles were exported as CSV files and visualized using Python libraries such as Seaborn (http://seaborn.pydata.org/) and Matplotlib (http://matplotlib.org/) to capture temporal trends in secondary-structure stability. Collectively, the integration of PCA, FEL, DSSP, dynamic cross-correlation (DCCM), and statistical analyses provided a comprehensive, statistically validated assessment of conformational plasticity, thermodynamic stability, and dynamic interaction mechanisms in the protein–peptide complexes.

### 2.9 MMPBSA

The Molecular Mechanics Poisson–Boltzmann Surface Area (MM/PBSA) method, one of the most widely used simulation approaches for calculating protein–ligand binding free energies, was employed to evaluate the binding affinities of TNF-alpha-peptide complexes. All calculations were performed using the gmx_MMPBSA package (https://valdes-tresanco-ms.github.io/gmx_MMPBSA/v1.6.0/) (Valdés-Tresanco et al., 2021), which integrates molecular mechanics and continuum solvent models to estimate the total binding free energy (ΔG_bind).

The binding free energy was computed as the difference between the free energy of the complex and that of its unbound components, using the following relationship:

$$\Delta G_{\text{bind}}=\Delta G_{\text{complex}}-\left(\Delta G_{\text{protein}}+\Delta G_{\text{ligand}}\right)$$

where ΔG_complex_ represents the total free energy of the TNF-alpha-peptide complex, and ΔG_protein_ and ΔG_ligand_ denote the free energies of the isolated TNF-alpha protein and the peptide, respectively.

Each free energy term (ΔG) was decomposed into potential energy (ΔE_MM) and solvation energy (ΔG_solv). The entropic contribution (−TΔS) was omitted to reduce computational cost, a common practice in comparative MM/PBSA analyses where relative binding energies are the focus. The total binding free energy can therefore be expressed as:

$$\Delta G_{\text{bind}}\approx \Delta G_{\text{potential}}+\Delta G_{\text{solvation}}$$

$$\Delta G_{\text{bind}}\approx \left(\Delta E_{\text{ele}}+\Delta E_{\text{vdW}}\right)+\left(\Delta G_{\text{GB}}+\Delta G_{\text{SA}}\right)$$

In this formulation, ΔE_ele and ΔE_vdW correspond to the electrostatic and van der Waals interaction energies derived from molecular mechanics calculations, respectively. The solvation free energy (ΔG_solv) comprises polar (ΔG_GB) and nonpolar (ΔG_SA) contributions. The polar solvation energy was computed using the Generalized Born (GB) implicit solvent model, while the nonpolar term was estimated based on the solvent-accessible surface area (SASA). Together, these components represent the total energy required for complex formation and solvation.

Finally, per-residue decomposition analysis was performed to identify the amino acid residues contributing most significantly to the overall binding free energy. These residues were considered critical for stabilizing the peptide–TNF-alpha complex and enhancing binding affinity of the designed inhibitors.

### 2.10 ADMET analysis

To assess the ADMET properties of the peptides, their sequences were converted into SMILES format using the PepSMI server (https://www.novoprolabs.com/tools/convert-peptide-to-smiles-string) and subsequently analyzed with pkCSM to predict key pharmacokinetic and toxicity parameters, including absorption, distribution, metabolism, excretion, and potential toxicity (Pires et al., 2015).

## 3 Results and discussion

### 3.1 Bioactive food-derived peptide curation

A comprehensive dataset of 200 bioactive peptides was systematically compiled from peer-reviewed literature, encompassing diverse biological activities including anti-inflammatory, antioxidant, immunomodulatory, antitumor, and anti-diabetic effects. This curated dataset highlights the remarkable therapeutic potential of dietary sources, including milk, marine organisms, legumes, vegetables, and fruits, as abundant reservoirs of health-promoting compounds. For each peptide, the amino acid sequence, source organism, and experimentally validated bioactivities were carefully documented, ensuring both accuracy and reliability as a foundation for subsequent *in silico* analyses. The curated peptides varied in length from 3 to 30 amino acids, representing a spectrum of functional properties. Short linear peptides are likely to be rapidly absorbed and exert immediate systemic effects, whereas longer sequences may adopt complex conformations that facilitate multifaceted biological interactions. This structural and functional diversity highlights the versatility of dietary proteins as precursors for bioactive fragments with potential applications across multiple therapeutic domains. Interestingly, despite the broad range of reported activities, only a few peptides have been explicitly studied for their anti-atherosclerotic potential, revealing a significant knowledge gap. Addressing this gap represents the novelty of the present computational approach, which aims to repurpose dietary peptides as modulators of cardiovascular disease pathways. After eliminating redundancies, the dataset was refined to 158 unique peptides. This curated library not only enhances the reliability of downstream computational analyses but also serves as a valuable resource for identifying nutraceutical candidates and peptide-based therapeutics targeting cardiovascular health.

### 3.2 Physicochemical properties of food-derived peptide

The physicochemical properties of 158 food-derived peptides were systematically evaluated using well-established web servers, including PepCalc and ProtParam. A comprehensive set of parameters was analyzed, encompassing molecular weight, net charge, isoelectric point (pI), instability index, half-life in mammalian cells, GRAVY (grand average of hydropathicity), and the distribution of negatively and positively charged residues. To ensure the identification of peptides with favorable biochemical stability and therapeutic relevance, a stringent multi-parameter filtering strategy was applied. Specifically, the pI was restricted to the range of 5.0–10.0, thereby selecting peptides with an appropriate ionization profile near physiological pH. The GRAVY index was constrained to −0.6 to +0.6, favoring peptides with a balanced amphipathic character that is essential for maintaining solubility while supporting hydrophobic contacts at protein–protein interfaces. The molecular weight window of 450–3,500 Da, and the charged distribution is constrained such that peptides should either display a balanced charge or a slight positive dominance to complement the largely neutral and polar environment of the TNF-alpha binding surface. Furthermore, only peptides predicted to be stable based on their instability index (<40) were retained.

Following the application of these rigorous thresholds, 19 high-confidence peptides (see Table 1) were shortlisted from an initial pool of 158 candidates. The adoption of strict criteria was particularly critical in the context of TNF-alpha inhibition, as peptides targeting this cytokine must be sufficiently small to access its shallow binding interface, amphipathic to sustain favorable protein–peptide contacts, and tuned in charge balance to optimize interactions with its neutral/polar residues. Only peptides meeting these combined physicochemical requirements are likely to demonstrate the structural robustness, amphipathic balance, and favorable electrostatics necessary to effectively modulate this cytokine. A detailed biological justification for each filtering parameter in relation to TNF-alpha interaction is summarized in Supplementary Table S1, highlighting the rationale for enforcing such rigorous selection rules. Furthermore, toxicity evaluation using a deep learning-based tool, ToxIBTL, confirmed that all shortlisted peptides were non-toxic. Collectively, this systematic filtering strategy ensured that the prioritized food-derived peptides represent the most promising candidates for further study.

**TABLE 1:** Physicochemical properties of food-derived peptide.

SL No.	Peptide sequence	Molecular weight	Net charge	pI	Number of residues	Extinction coefficient	Half-life	Instability index	Grand average of hydropathicity (GRAVY)	Total number of negatively charged residues	Total number of positively charged residues	Toxicity
1	GVDYVRFF	1,002.12 g/mol	0	6.59	8	1280 M^−1^cm^−1^	30 h	−10.04 (stable)	0.537	1	1	Non-Toxin
2	ALNKTHLIQTK	1,266.49 g/mol	2.1	10.74	11	0 M^−1^cm^−1^	4.4 h	30.62 (stable)	−0.5	0	2	Non-Toxin
3	VVVLRDGAVQQLGTPR	1,707.97 g/mol	1	9.5	16	0 M^−1^cm^−1^	100 h	18.12 (stable)	0.225	1	2	Non-Toxin
4	IPDAHPVK	876.01 g/mol	0.1	7.8	8	0 M^−1^cm^−1^	20 h	1.99 (stable)	−0.412	1	1	Non-Toxin
5	IAGPAGPRGPSGPA	1,204.34 g/mol	1	9.75	14	0 M^−1^cm^−1^	20 h	39.11 (stable)	−0.243	0	1	Non-Toxin
6	GPEGPMGL	756.87 g/mol	−1	5.5	8	0 M^−1^cm^−1^	30 h	21.05 (stable)	−0.27	1	0	Non-Toxin
7	GPGLM	473.59 g/mol	0	5.52	5	0 M^−1^cm^−1^	30 h	8.00 (stable)	0.6	0	0	Non-Toxin
8	PKKVV	569.74 g/mol	2	10	5	0 M^−1^cm^−1^	20 h	−8.98 (stable)	−0.2	0	2	Non-Toxin
9	MEPLGQG	730.83 g/mol	−1	5	7	0 M^−1^cm^−1^	30 h	36.09 (stable)	−0.529	1	0	Non-Toxin
10	QCQCAVEGGL	1,007.15 g/mol	−1.1	6.2	10	0 M^−1^cm^−1^	0.8 h	−1.28 (stable)	0.35	1	0	Non-Toxin
11	ALGTWK	674.79 g/mol	1	8.08	6	5690 M^−1^cm^−1^	4.4 h	−30.87 (stable)	−0.05	0	1	Non-Toxin
12	KIWHHTF	968.11 g/mol	1.2	8.71	7	5690 M^−1^cm^−1^	1.3 h	37.17 (stable)	−0.557	0	1	Non-Toxin
13	TVNLAYY	842.93 g/mol	0	5.18	7	2560 M^−1^cm^−1^	7.2 h	26.20 (stable)	0.429	0	0	Non-Toxin
14	LPHSGY	672.73 g/mol	0.1	7.75	6	1280 M^−1^cm^−1^	5.5 h	26.28 (stable)	−0.583	0	0	Non-Toxin
15	FGASTRGA	765.81 g/mol	1	9.75	10	0 M^−1^cm^−1^	1.1 h	−23.09 (stable)	−0.05	0	1	Non-Toxin
16	GAHAGPTWNPISIGISFMGNYMNR	2,591.92 g/mol	1.1	8.75	24	6970 M^−1^cm^−1^	30 h	1.42 (stable)	−0.158	0	1	Non-Toxin
17	PPYCTIVPFGIFGTNYR	1,945.24 g/mol	0	8.9	19	2560 M^−1^cm^−1^	20 h	28.81 (stable)	0.218	0	1	Non-Toxin
18	IAYKPAG	718.84 g/mol	1	9.75	7	1280 M^−1^cm^−1^	20 h	25.31 (stable)	0.129	0	1	Non-Toxin
19	LYTPH	629.7 g/mol	0.1	7.75	5	1280 M^−1^cm^−1^	5.5 h	−8.98 (stable)	−0.6	0	0	Non-Toxin

### 3.3 Evaluation of cell-penetrating, anti-inflammatory property, and bioactivity score prediction of peptides

A total of 19 food-derived peptides were initially shortlisted based on favorable physicochemical properties. To further evaluate their therapeutic relevance, an *in silico* screening was conducted to assess bioactivity score, anti-inflammatory potential (AIP), and cell-penetrating capacity (CPP). These parameters serve as important indicators of therapeutic efficacy, where the bioactivity score reflects the probability of biological activity, AIP prediction identifies potential anti-inflammatory effects, and CPP estimation indicates the ability of peptides to penetrate cellular membranes.

From Table 2, it was observed that the predicted bioactivity values for the 19 peptides ranged between 0.068 and 0.91. Applying a threshold of ≥0.5, only eight peptides were initially considered promising candidates. This cutoff ensured the selection of sequences with higher potential to interact with relevant biological targets. Subsequently, all shortlisted peptides were further analyzed for their anti-inflammatory activity using the AIPpred server. Interestingly, with the exception of two peptides, nearly all food-derived peptides were predicted to possess anti-inflammatory properties. This finding is particularly important, as modulation of inflammation is central to the therapeutic strategy under investigation. Finally, the selected peptides were evaluated for their cell-penetrating property (CPP). None of the sequences were predicted as CPP, suggesting that their therapeutic action is likely mediated through extracellular or membrane-bound interactions rather than direct intracellular targeting, which aligns with the targeting of cytokines such as TNF-alpha. Although this may limit their independent use as intracellular delivery agents, it does not diminish their potential value as anti-inflammatory peptides. overall, this integrative analysis narrowed down the pool from nineteen to seven peptides that satisfied the dual criteria of high bioactivity (≥0.5) and positive AIP prediction. The shortlisted peptides were subjected to molecular docking and dynamics simulations, underscoring the value of multi-parameter *in silico* screening in prioritizing only the most promising candidates for resource-intensive analyses.

**TABLE 2:** Different biological properties of the food-derived peptides.

SL No.	Peptide sequence	Bioactivity score	AIP	CPP	Mutation
1	IAGPAGPRGPSGPA	0.76	AIP	Non-CPP	No mutation
2	ALNKTHLIQTK	0.106	Non-AIP	Non-CPP	No mutation
3	VVVLRDGAVQQLGTPR	0.121	AIP	Non-CPP	No mutation
4	IPDAHPVK	0.368	AIP	Non-CPP	No mutation
5	GVDYVRFF	0.857	AIP	Non-CPP	No mutation
6	GPEGPMGL	0.855	AIP	Non-CPP	No mutation
7	GPGLM	0.91	Non-AIP	Non-CPP	No mutation
8	PKKVV	0.068	AIP	Non-CPP	No mutation
9	MEPLGQG	0.283	AIP	Non-CPP	No mutation
10	KIWHHTF	0.732	AIP	Non-CPP	No mutation
11	ALGTWK	0.552	AIP	Non-CPP	No mutation
12	QCQCAVEGGL	0.607	AIP	Non-CPP	No mutation
13	TVNLAYY	0.101	AIP	Non-CPP	No mutation
14	LPHSGY	0.409	AIP	Non-CPP	No mutation
15	FGASTRGA	0.307	AIP	Non-CPP	No mutation
16	GAHAGPTWNPISIGISFMGNYMNR	0.285	AIP	Non-CPP	No mutation
17	PPYCTIVPFGIFGTNYR	0.781	AIP	Non-CPP	No mutation
18	IAYKPAG	0.465	AIP	Non-CPP	No mutation
19	LYTPH	0.331	AIP	Non-CPP	No mutation

### 3.4 Structural integrity assessment of food-derived peptides

The three-dimensional (3D) structures of the shortlisted food-derived peptides were predicted using PEP-FOLD4, a state-of-the-art fragment-based modeling approach optimized for short therapeutic peptides. The server employs a sophisticated workflow that combines a structural alphabet (SA) framework with a sOPEP coarse-grained force field to efficiently sample conformational space and generate five potential models for each peptide. The lowest-energy conformation was selected for all subsequent analyses. PEP-FOLD4 is uniquely suited for this task due to its use of shape descriptors and the inclusion of Debye–Hückel formalism to account for variations in pH and ionic strength.

**FIGURE 3:**
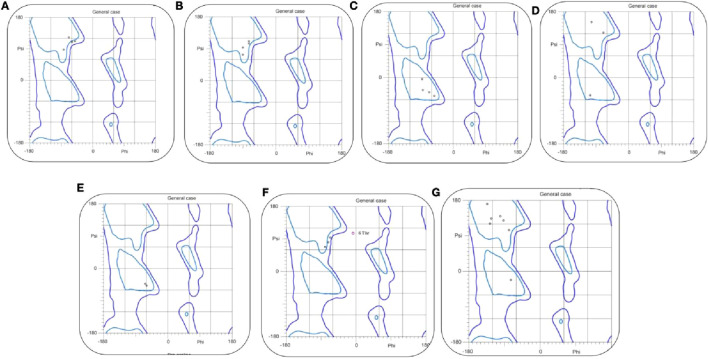
The Ramachandran plots of TNF-alpha inhibitory peptides. **(A–G)** illustrate backbone φ/ψ angles for Seq1–Seq7 and the reference complex, respectively. Blue contours mark favored/allowed regions, and black dots indicate residue conformations, confirming that all peptides adopt sterically allowed geometries.

Following prediction, the models were subjected to rigorous stereochemical validation using the MolProbity server, with results visualized through Ramachandran plots (Figure 3). This analysis, a crucial quality control step, confirmed the correct stereochemical configuration of backbone torsion angles (ϕ and ψ) for each peptide. The plots demonstrate that the residues are positioned predominantly within the most favored and additionally in the allowed regions, with only a few falling into outlier zones. This distribution indicates that the predicted models are not only theoretically stable but also physically feasible and biologically relevant. Such high structural fidelity provides strong confidence in their suitability for downstream computational analyses, including molecular docking and molecular dynamics simulations, ensuring that the modeled structures can faithfully engage in biologically meaningful interactions.

### 3.5 Rational redesign of TNFR1 binding motifs as potential TNF-alpha inhibitory peptides

**FIGURE 4:**
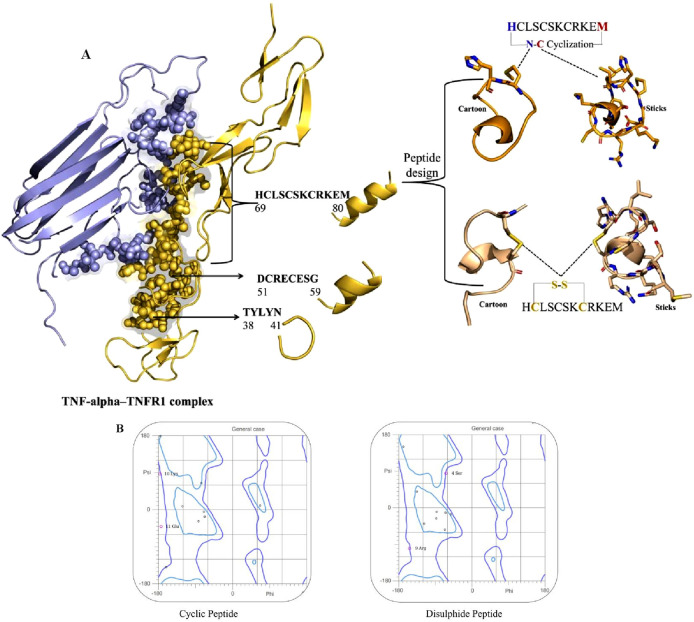
**(A)** Structural mapping and design of TNF-alpha–derived inhibitory peptides. Left: Crystal structure of the TNF-alpha–TNFR1 complex, with TNF-alpha shown in lavender and TNFR1 in golden. TNF-alpha residues that directly interact with the TNFR1 receptor are highlighted as spheres, indicating key binding sites. Right: Peptide design based on the TNF-alpha epitope HCLSCSKCRKEM (residues 69–80). Two cyclic variants are illustrated: top, N–C terminal cyclization; bottom, intramolecular disulfide (S–S) bridge between cysteine residues. For each variant, cartoon and stick representations depict the stabilized conformations used for computational analyses. **(B)** The Ramachandran plot of cyclic and disulfide peptides.

To identify potential therapeutic peptides, the molecular interface of the TNF-alpha–TNFR1 complex (PDB ID: 7KPB) was first analyzed to map the critical binding motifs. This structural mapping revealed three short segments of TNFR1 that directly interact with TNF-alpha, namely residues 38–41 (TYLYN), 51–59 (DCRECESG), and 69–80 (HCLSCSKCRKEM) (Figure 4A). These receptor-derived fragments represent key interface motifs mediating TNF-alpha recognition and were therefore extracted as candidate peptide templates for further evaluation. Following this initial identification, these sequences were evaluated for anti-inflammatory potential (AIP), cell-penetrating property (CPP), and bioactivity score (Supplementary Table S2). While all three peptides showed anti-inflammatory activity, the shorter sequences, TYLYN and DCRECESG, exhibited low bioactivity scores, limiting their therapeutic suitability. Crucially, none of the three peptides displayed CPP activity, which suggested their functional role would be restricted to extracellular or receptor-level interactions.

Among the three receptor-derived motifs, HCLSCSKCRKEM (residues 69–80) emerged as the most promising candidate. In addition to its positive anti-inflammatory potential and favorable bioactivity score, its extended sequence length provides a broader interaction interface, which increases the likelihood of stable binding with TNF-alpha. This combination of properties led to its selection as the lead TNFR1-derived inhibitory peptide for rational redesign and optimization. To improve the drug-like properties and structural robustness, two modified analogues were rationally designed. The first, a cyclic analogue, was generated through N–C terminal (head-to-tail) cyclization, a strategy that eliminates the free N- and C-termini, thereby enhancing resistance to enzymatic degradation. This induced conformational pre-organization, which is highly advantageous as it minimizes the entropic penalty incurred upon target binding, resulting in improved affinity and specificity. While a disulfide-stabilized analogue was designed by incorporating intramolecular disulfide bridges. These covalent linkages enhance structural stability by constraining the backbone and preserving the bioactive conformation essential for effective interaction with the target protein. The compact, stabilized structure of both modified analogues is reported to confer cell-penetrating potential (Troeira Henriques et al., 2025). The three-dimensional structures of these redesigned analogues were subsequently modeled and refined using the PEPstrMOD server, providing accurate conformational representations for downstream computational analyses.

The stereochemical quality of the modeled peptides was rigorously validated using Ramachandran plot analysis, a key component of the MolProbity server comprehensive assessment (Figure 4B). The cyclic peptide showed the majority of residues clustered within highly favored regions of the φ–ψ torsional space, with Lys10 and Glu11 positioned in energetically stable conformations, reflecting the stabilizing influence of terminal cyclization. Likewise, the disulfide-stabilized peptide exhibited a similarly favorable distribution, with residues such as Ser4 and Arg9 occupying permissible regions that are consistent with natural backbone flexibility. The introduction of disulfide linkages reinforced structural compactness while preserving stereochemical integrity. Overall, the structural validation of both cyclic and disulfide-stabilized variants confirmed their conformational stability, minimal steric clashes, and favorable stereochemical profiles. These findings establish that the rationally designed peptides are structurally viable candidates for downstream docking and molecular simulation analyses. Collectively, the identification of TNF-alpha interacting segments, coupled with rational peptide redesign and stereochemical validation, provides strong evidence that cyclic and disulfide-stabilized peptides can serve as promising inhibitors of TNF-alpha, thereby justifying their selection for computational and functional evaluations.

### 3.6 Molecular docking of food-derived and designed peptides

To evaluate the binding potential of the selected peptides against TNF-alpha, a total of seven food-derived peptides and two rationally redesigned TNFR1-derived peptides (cyclic and disulfide-stabilized variants of HCLSCSKCRKEM) were subjected to molecular docking analyses using two independent platforms, HPEPDOCK and HADDOCK. Employing this dual-platform strategy ensured cross-validation of docking outcomes, thereby strengthening the reliability of the predictions. Peptides exhibiting high docking scores in both tools (above −170 kcal/mol in HPEPDOCK and better than −75 kcal/mol in HADDOCK) were shortlisted as top candidates for further investigation, as these thresholds correspond to strong binding affinity and optimal structural complementarity with TNF-alpha. Among the food-derived peptides, several sequences exhibited strong binding affinities. In particular, GVDYVRFF (marine), KIWHHTF (marine), and PPYCTIVPFGIFGTNYR (wheat) exhibited the most favorable docking scores, highlighting their potential as TNF-alpha modulators. Specifically, GVDYVRFF scored −177.899 in HPEPDOCK and −76.6 ± 0.8 in HADDOCK, KIWHHTF scored −194.936 and −88.9 ± 4.1, and PPYCTIVPFGIFGTNYR achieved −198.923 and −96.5 ± 5.1, respectively. These results indicate that peptides from both marine and plant sources can form stable and energetically favorable interactions with TNF-alpha, reflecting the therapeutic potential of structurally diverse dietary peptides.

The rationally redesigned TNFR1-derived peptide analogues also displayed notable stability in docking evaluations. The cyclic HCLSCSKCRKEM peptide achieved a docking score of −178.913 in HPEPDOCK and −77.7 ± 2.3 in HADDOCK, while the disulfide-stabilized variant yielded −171.932 and −75.8 ± 2.7, respectively. Although their scores were slightly less favorable compared to the top-performing food-derived peptides, both redesigned analogues maintained consistently strong interactions with TNF-alpha. Importantly, their docking results were substantially superior to the standard native ligand, which registered only −44.0 ± 1.3 in HADDOCK, thereby confirming the enhanced binding potential and therapeutic relevance of the redesigned peptides. The results are displayed in Table 3.

**TABLE 3:** Docking score of the food-derived and redesigned peptide against TNF-alpha protein.

Peptide sequence	Docking score HPEPDOCK	HADDOCK
GVDYVRFF	−177.899	−76.6 ± 0.8
IAGPAGPRGPSGPA	−167.567	−73.8 ± 2.0
GPEGPMGL	−131.349	−55.6 ± 0.9
ALGTWK	−153.205	−69.9 ± 3.0
QCQCAVEGGL	−152.937	−62.5 ± 1.5
KIWHHTF	−194.936	−88.9 ± 4.1
PPYCTIVPFGIFGTNYR	−198.923	−96.5 ± 5.1
HCLSCSKCRKEM (Cyclic peptide)	−178.913	−77.7 ± 2.3
HCLSCSKCRKEM (Disulphide cyclic peptide)	−171.932	−75.8 ± 2.7
Standard	—	−44.0 ± 1.3

Overall, these docking analyses demonstrate that both food-derived and rationally designed TNFR1-derived peptides possess strong TNF-alpha binding capabilities. The selected food-derived peptides, GVDYVRFF and KIWHHTF from marine sources, and PPYCTIVPFGIFGTNYR from wheat, highlight the therapeutic relevance of diverse dietary origins, while the cyclic and disulfide-stabilized TNFR1-derived analogues confirm that structural optimization strategies can enhance stability and interaction potential. Based on these results, these five peptides were prioritized for subsequent molecular dynamics simulations to evaluate their temporal stability, conformational flexibility, and functional interactions under dynamic conditions. These findings underscore the value of combining dietary peptide screening with structure-guided redesign to generate effective TNF-alpha inhibitors for potential therapeutic development.

#### 3.6.1 Molecular interactions of food-derived and redesigned peptides

**FIGURE 5:**
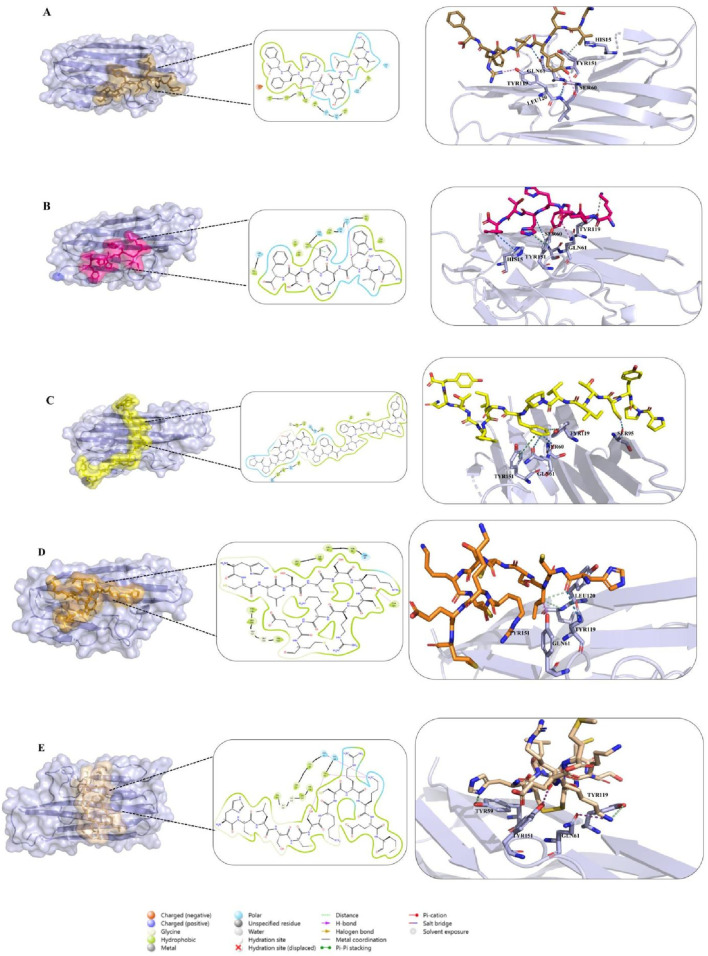
**(A–C)** Docking interactions of food-derived peptides with TNF-alpha. Binding interactions of food-derived peptides with TNF-alpha (light blue surface). **(A)** Seq1 (sand), **(B)** Seq6 (hot pink), and **(C)** Seq7 (yellow). The left show the TNF-alpha surface representation with the peptide binding interface highlighted; the middle insets display 2D interaction maps illustrating hydrogen bonds and hydrophobic contacts; and the right present close-up 3D views of key interacting residues. **(D,E)** Docking interactions of designed peptides with TNF-alpha. Binding interactions of rationally designed peptides with TNF-alpha (light blue surface). **(D)** Cyclic peptide (orange) and **(E)** disulfide-stabilized peptide (wheat) are shown. The left display the TNF-alpha surface representation with each peptide at its binding interface; the middle insets illustrate 2D interaction maps indicating hydrogen bonds, hydrophobic, and polar contacts; and the right present close-up 3D views of key interacting residues.

To gain deeper insight into the molecular determinants of TNF-alpha inhibition, three food-derived peptides (Seq1: GVDYVRFF, Seq6: KIWHHTF, and Seq7: PPYCTIVPFGIFGTNYR) and two redesigned TNFR1-derived analogues (cyclic and disulfide-stabilized HCLSCSKCRKEM) were analyzed for their interaction profiles with TNF-alpha (PDB ID: 2AZ5). Docking validation using both HPEPDOCK and HADDOCK 2.4 yielded consistent binding poses, underscoring the reliability of the predicted peptide–protein complexes Figures 5A–E. Table 4 summarizes the key residues, interaction types, and characteristic binding features for each peptide. The food-derived peptide GVDYVRFF (Seq1) exhibited strong binding affinity within the TNF-alpha interaction pocket through a network of hydrogen bonds and hydrophobic interactions. Polar contacts were formed with GLN61, SER60, GLN149, and HIS15, complemented by hydrophobic interactions involving TYR115, PRO117, LEU63, TYR59, and TYR151. Notably, TYR119 and LEU120 participated in both hydrogen bonding and hydrophobic interactions, acting as dual anchoring residues that reinforced complex stability.

**TABLE 4:** Comparative summary of key TNF-alpha–peptide interactions.

Peptide ID	Sequence	Key polar interactions	Key hydrophobic residues	Dual/Special interactions	Distinctive feature
Seq1	GVDYVRFF	GLN61, SER60, GLN149, HIS15	TYR59, LEU63, TYR115, PRO117, TYR151	TYR119 and LEU120-dual anchoring	Strong aromatic–aliphatic complementarity
Seq6	KIWHHTF	GLN61, HIS15, SER60, GLN149	VAL13, LEU36, TYR59, TYR119, ILE155	TYR151- π–π stacking and hydrophobic interaction	Compact binding with stacking-driven stability
Seq7	PPYCTIVPFGIFGTNYR	SER60, GLN61, SER95, ASN92	TYR59, LEU94, TYR119, LEU120, ILE155	TYR151 – π–π stacking	Multisite anchoring, strong aromatic contacts
Cyclic	HCLSCSKCRKEM	SER60	LEU57, TYR59, TYR119, LEU120, VAL123, ILE155, LEU157	TYR151 – dual H-bond + hydrophobic	Pre-organized backbone; high rigidity
Disulfide	HCLSCSKCRKEM (Cys2–Cys8)	GLN61, GLN149	TYR59, LEU57, TYR119, LEU120, VAL123	TYR151 – dual anchoring	Disulfide constraint; enhanced persistence

Similarly, in Figure 5B, molecular interaction analysis of the other food-derived peptide KIWHHTF (Seq6) with TNF-alpha demonstrated a compact and stable binding conformation. This conformation was stabilized primarily by polar contacts with key residues GLN61, HIS15, SER60, and GLN149, and reinforced by hydrophobic interactions involving VAL13, LEU36, TYR59, LEU63, TYR119, and ILE155. The residue TYR151 contributed both *via* π–π stacking and hydrophobic interactions, providing additional stability to the peptide’s presence at the binding interface.

The docking analysis of PPYCTIVPFGIFGTNYR (Seq7) (Figure 5C) demonstrated a robust binding mode stabilized by multiple hydrogen bonds and hydrophobic forces. Key polar contacts were established with SER60, GLN61, SER95, and ASN92. Extensive hydrophobic contacts anchored the peptide *via* TYR59, LEU93, LEU94, ALA96, TYR119, LEU120, and ILE155. The protein residue TYR151 also contributed significantly through pi-pi stacking and hydrophobic interactions. Collectively, these combined polar, hydrophobic, and pi-pi stacking forces underscore the high binding affinity and structural robustness of the Seq7-TNF-alpha complex.

The redesigned constrained peptides exhibited highly stable binding modes (Figures 5D,E). The cyclic analogue, HCLSCSKCRKEM (with the N- and C-termini covalently linked to form a cyclic scaffold) exhibited a compact, conformationally constrained architecture that pre-organized key residues for target binding. A central polar interaction with SER60 served as a primary anchoring point, while a hydrophobic network involving LEU57, TYR59, TYR119, LEU120, VAL123, ILE155, and LEU157 enhanced van der Waals packing. TYR151 provided dual hydrogen bonding and hydrophobic interactions, promoting exceptional structural complementarity and rigidity.

Similarly, the disulfide-stabilized peptide (Figure 5E) (HCLSCSKCRKEM with a Cys2–Cys8 bridge) exploited its rigid, pre-organized conformation to achieve an optimal fit within the TNF-alpha binding pocket. This analogue was anchored through polar interactions with GLN61 and GLN149, supplemented by extensive hydrophobic packing involving TYR59, LEU57, TYR119, LEU120, and VAL123. The multifunctional TYR151 residue further reinforced the complex through concurrent hydrogen-bonding and hydrophobic contacts. Collectively, both constrained peptide architectures exhibited high-affinity, conformationally stable, and multi-residue engagement profiles, underscoring their structural resilience and inhibitory potential. Overall, the docking studies revealed that both the food-derived and redesigned peptides consistently engaged with the key functional residues of TNF-alpha, including LEU57, TYR59, SER60, GLN61, TYR119, LEU120, GLY121, GLY122, and TYR151, thereby establishing a robust and well-defined interaction profile within the binding groove. Among these, TYR119 emerged as particularly significant, consistent with the observations of (He et al., 2005), who demonstrated that rotation of its χ^1^ angle facilitates ligand accommodation and promotes TNF-alpha dimer formation, and was likewise identified by (Shah and Arumugam, 2024), as a crucial determinant of ligand binding. Notably, TYR119, LEU120, and TYR151 exhibited a dual interaction pattern across all peptide complexes, contributing simultaneously through hydrophobic stacking and hydrogen-bonding contacts. The consistent engagement of these residues, along with the surrounding stabilizing interactions, underscores the strong potential of both food-derived and redesigned peptides as structurally resilient and functionally effective TNF-alpha inhibitors.

### 3.7 Exploring the stability and flexibility of peptide–TNF-alpha complexes through MD simulations

To complement and validate the docking analyses, molecular dynamics (MD) simulations were conducted for the five prioritized peptides: three food-derived sequences (GVDYVRFF, KIWHHTF, PPYCTIVPFGIFGTNYR) and two rationally redesigned analogues (cyclic and disulfide-stabilized HCLSCSKCRKEM). Unlike static docking models, MD simulations provide a time-resolved representation of biomolecular interactions, enabling the assessment of structural stability, conformational flexibility, and binding persistence under near-physiological conditions. By monitoring key parameters such as root-mean-square deviation (RMSD), root-mean-square fluctuation (RMSF), hydrogen bond occupancy, and radius of gyration (Rg), the stability of the complexes was systematically evaluated. This dynamic approach not only confirmed the robustness of peptide binding but also revealed critical insights into the flexibility of interacting residues and the conformational adaptability of the cyclic peptides within the TNF-alpha binding pocket.

#### 3.7.1 RMSD-based stability assessment of food-derived and interface peptide–TNF-alpha complexes

**FIGURE 6:**
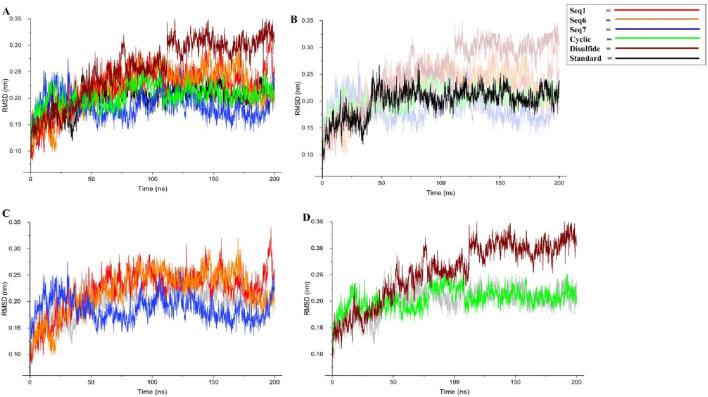
Backbone RMSD analysis of TNF-alpha–peptide complexes over 200 ns MD simulations. **(A)** presents the global RMSD profiles for all systems, enabling direct comparison between the standard reference, three food-derived peptides (Seq1, Seq6, Seq7), and two rationally designed peptides (cyclic, disulfide). **(B)** isolates the standard TNF-alpha complex, **(C)** displays only the food-derived peptide complexes, and **(D)** highlights the designed cyclic and disulfide inhibitors.

The root mean square deviation (RMSD) profiles were evaluated to examine the stability of TNF-alpha in complex with food-derived peptides, redesigned peptides, and the standard inhibitor over a 200 ns molecular dynamics simulation (Figure 6; Table 5). To improve clarity, the data were represented in four panels: (A) all six peptide complexes, (B) the standard inhibitor, (C) the three food-derived peptides (Seq1, Seq6, Seq7), and (D) the redesigned peptides (cyclic and disulfide). The standard inhibitor maintained a stable trajectory with an average RMSD of 0.199 ± 0.02 nm, serving as a benchmark for comparison. Among the food-derived peptides, Seq7 exhibited the lowest RMSD (0.186 ± 0.02 nm), which was slightly lower than the standard. Interestingly, after ∼30 ns, Seq7 showed a gradual downward trend, stabilizing even further as the simulation progressed, suggesting strong conformational adaptability within the TNF-alpha binding pocket. By contrast, Seq1 (0.221 ± 0.04 nm) and Seq6 (0.219 ± 0.04 nm) displayed nearly identical RMSD values and trends throughout the trajectory, remaining consistently close to the standard, which indicates that both peptides similarly adapt to the TNF-alpha structure. For the redesigned peptides, the cyclic peptide (0.204 ± 0.02 nm) demonstrated a trajectory nearly overlapping with the standard, with only minor fluctuations, underscoring the stabilizing influence of cyclization on conformational rigidity. On the other hand, the disulfide-stabilized peptide (0.256 ± 0.05 nm) showed a relatively higher RMSD and notable spikes after ∼50 ns, indicating transient conformational adjustments before stabilizing toward the latter part of the simulation. Despite these fluctuations, its RMSD remained within an acceptable range (<0.30 nm), confirming the overall structural stability of the complex. In conclusion, these results highlight that Seq7 and the Cyclic peptide demonstrate stability profiles highly comparable to the standard inhibitor, positioning them as strong candidates for further development. The observed behavior also emphasizes the advantages of Cyclic peptides in maintaining structural integrity and the unique stability of Seq7, which even outperformed the standard. While Seq1, Seq6, and the disulfide peptide maintained slightly higher RMSD values, they still exhibited stable trajectories, validating their potential as TNF-alpha binders.

**TABLE 5:** Average values of structural parameters for TNF–alpha-food-derived and designed complexes over the 200 ns MD simulation. The table presents the average values of RMSD, RMSF, radius of gyration, and H-bond for both protein–peptide complexes and their respective standard (native ligand), providing insights into their structural stability and compactness during the simulation period.

SL No.	Average value		Food-derived peptide			Redesigned peptide	
		Standard	Seq1	Seq6	Seq7	Cyclic peptide	Disulfide bridge peptide
1	RMSD	0.199 ± 0.02	0.221 ± 0.04	0.219 ± 0.04	0.186 ± 0.02	0.204 ± 0.02	0.256 ± 0.05
2	RMSF	0.103 ± 0.05	0.108 ± 0.06	0.124 ± 0.07	0.102 ± 0.06	0.106 ± 0.05	0.119 ± 0.07
3	H-bond	0.674 ± 0.84	1.395 ± 1.00	1.557 ± 1.48	1.543 ± 1.27	3.058 ± 1.42	1.656 ± 1.57
4	Rg	1.546 ± 0.006	1.565 ± 0.01	1.545 ± 0.009	1.546 ± 0.008	1.538 ± 0.007	1.534 ± 0.01

##### 3.7.1.1 Dynamic reaction patterns of the peptide-receptor conjugates

**FIGURE 7:**
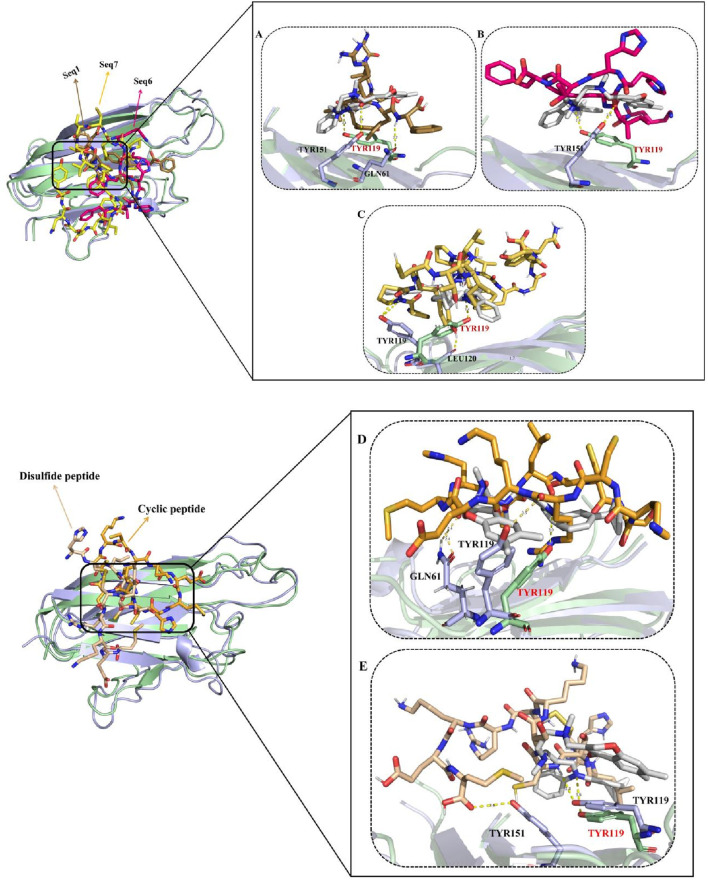
Panels **(A–C)** show structural superimposition of the TNF-alpha–ligand standard complex with peptide-bound complexes. The TNF-alpha backbone appears pale green for the standard complex and light blue for peptide-bound states. Peptides are displayed in distinct colors: Seq1 (sand), Seq6 (pink), and Seq7 (yellow). Panels **(D)** and **(E)** show similar overlays for the cyclic (orange) and disulfide-stabilized (wheat) peptides. The figure highlights comparable binding orientations and stabilization patterns of all peptides within the TNF-alpha binding groove, with hydrogen bonds represented as yellow dashed lines.

To further validate the inhibitory mechanisms of the peptide complexes, a comparative molecular dynamics (MD) analysis was performed by superimposing the trajectories of food-derived and designed peptide–TNF-alpha complexes with that of the standard ligand–TNF-alpha complex. This frame-wise evaluation over a 200 ns simulation window enabled the identification of temporally resolved polar contacts and their correspondence with the established inhibitory hotspot residues of TNF-alpha (Figure 7). The detailed interactions, including key residues, dominant interaction types, interaction timeframes, and observed behavior, are summarized in Table 6, providing a concise comparative view of peptide binding dynamics.

**TABLE 6:** Dynamic interaction profiles of peptide–TNF-alpha complexes from molecular dynamics simulations.

Peptide ID	Interaction timeframe (ns)	Key residue involved	Interaction type	Observed behavior/stability
Standard	55.6	TYR119	Polar contact	Persistent interaction; stable occupancy of inhibitory site
Seq1 (Food-derived peptide)	0–20 (initial)	GLN61, TYR119 (transient), LEU120, TYR151	Polar contact	Alternative stabilization; maintains proximity to TYR119 hotspot
Seq6 (Food-derived peptide)	56.0	TYR151 (main anchor)	Polar contact	Alternative stabilization; anchors within the inhibitory region
Seq7 (Food-derived peptide)	55–57.1	TYR119, LEU120	Polar contact	High Fidelity; closely mimics standard ligand’s TYR119 stabilization
Cyclic	53.3–56.9	GLN61, TYR119	Polar contact	High Fidelity; compact structure ensures consistent TYR119 engagement
Disulfide	55.0–56.8	TYR59, SER60 TYR119 TYR151	Polar contact	Dynamic behaviour, but maintains TYR119 engagement

The standard ligand established a persistent polar interaction with TYR119 at ∼55.6, consistent with its established role as a key residue mediating TNF-alpha inhibition (He et al., 2005). This stable interaction effectively occupies the receptor-binding site, thereby preventing subsequent ligand engagement and downstream signal transduction. For the food-derived peptides, the analysis revealed distinct but effective binding strategies. The Seq1 peptide initially (0–20 ns) engaged LEU120, TYR151, and GLN61, before stabilizing with TYR151 and GLN61. While its interaction with TYR119 was transient, its close proximity to the inhibitory pocket confirms it can maintain binding-site fidelity. Similarly, Seq6 used an alternative anchoring strategy by interacting with TYR151 at ∼56 ns, a residue located within the same inhibitory region. Finally, the Seq7 peptide most closely mimicked the standard ligand, consistently forming polar bonds with TYR119 and LEU120 between 55 and 57.1 ns, effectively capitalizing on the crucial TYR119 mediated stabilization mechanism to physically block receptor binding. The analysis of the designed peptides further reinforced our findings.

Analysis of the designed analogues further reinforced these findings. The cyclic peptide formed stable polar interactions with GLN61 and TYR119 between ∼53.3 and 56.9 ns, successfully targeting the primary inhibitory hotspot. Its compact, head-to-tail cyclized structure imposed conformational rigidity that minimized structural drift, ensuring continuous engagement with TYR119 throughout the simulation. Conversely, the disulfide-stabilized peptide displayed greater conformational flexibility, transitioning from initial contacts with SER60, TYR119, and TYR151 to more stable interactions with TYR59 and TYR119 between ∼55 and 56.8 ns. This adaptive binding behavior enabled it to maintain inhibitory alignment within the binding pocket despite localized positional adjustments.

Overall, the docking studies revealed that all peptides consistently engaged the critical residues (TYR119, LEU120, and TYR151), and these interactions were further reaffirmed by the molecular dynamics simulation analysis. These residues exhibited dual interaction patterns across both docking and dynamic analyses, ensuring robust and persistent occupancy of the TNF-alpha binding pocket and highlighting the structural resilience of the peptide candidates. The mechanistic reliability of these binding modes across all peptides was further supported by persistent interactions with additional inhibitory residues (SER60 and GLN61), physically blocking receptor association and preventing downstream signaling. While Seq1 and Seq6 adopted alternative stabilizing strategies, their engagement within the same inhibitory region as the standard ligand maintained critical contacts. In contrast, Seq7, along with the cyclic and disulfide-stabilized peptides, demonstrated superior binding fidelity and structural stability, closely recapitulating the native ligand inhibitory mode and enhancing their capacity to disrupt TNF-alpha–mediated signaling.

#### 3.7.2 Flexibility profiling of TNF-alpha active site and loop regions

**FIGURE 8:**
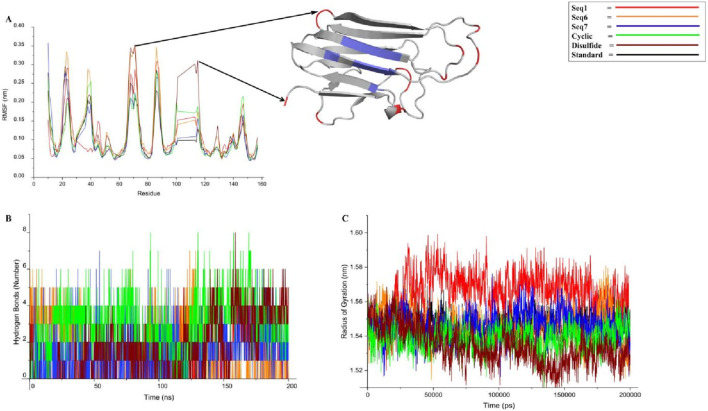
This figure presents a concise analysis of the peptide-TNF-alpha complexes stability over a 200 ns molecular dynamics simulation. **(A)** displays the RMSF (Root Mean Square Fluctuation) profile, which visually confirms the protein inherent dynamics: the β-sheet regions (blue) remain rigid, while the flexible loops (red) show expected fluctuation across all peptide complexes. **(B)** tracks the number of hydrogen bonds over time. Finally, **(C)** presents the Radius of Gyration (Rg) profiles.

The Root Mean Square Fluctuation (RMSF) analysis was performed to evaluate residue-level flexibility of TNF-alpha in complex with the standard ligand, three food-derived peptides (Seq1, Seq6, Seq7), and redesigned peptides (cyclic and Disulfide) over the course of the 200 ns MD simulation (Figure 8A; Table 5). The RMSF profiles revealed that the majority of fluctuations were localized within the loop regions, as highlighted in red in the structural mapping, whereas the active site residues (depicted in blue) remained comparatively stable with minimal deviations. This observation indicates that peptide binding did not disrupt the conformational stability of the critical functional pocket of TNF-alpha. The calculated average RMSF values were as follows: standard (0.103 ± 0.05 nm), Seq1 (0.108 ± 0.06 nm), Seq6 (0.124 ± 0.07 nm), Seq7 (0.106 ± 0.06 nm), cyclic peptide (0.106 ± 0.05 nm), and disulfide peptide (0.119 ± 0.07 nm). Among these, Seq6 and the disulfide peptide displayed slightly higher fluctuations, suggesting localized flexibility within certain loop regions, whereas Seq7 and the cyclic peptide showed RMSF values closely matching the standard ligand, highlighting their ability to maintain structural stability at the binding interface. Importantly, residues forming the TNF-alpha active site remained consistently rigid across all peptide complexes, indicating that the peptides do not induce destabilization of the critical binding pocket. This preservation of active site rigidity is particularly notable for Seq7 and the cyclic analogue, supporting their potential for high-affinity inhibition by maintaining precise complementarity with key functional residues. Collectively, these RMSF results suggest that while peripheral loop regions undergo natural dynamic motions, the peptide–TNF-alpha complexes, especially those involving Seq7 and the cyclic peptide, maintain stable engagement with the active site, ensuring conformational integrity essential for inhibitory activity. These findings reinforce the observations from RMSD and molecular interaction analyses, highlighting the structural robustness and functional relevance of the prioritized peptides.

#### 3.7.3 Hydrogen bond analysis

Hydrogen bonding plays a critical role in maintaining the stability and specificity of protein–ligand interactions. The hydrogen bond dynamics for all peptide–TNF-alpha complexes and the standard ligand were analyzed over the 200 ns simulation trajectory (Figure 8B; Table 5). The average number of hydrogen bonds observed for the standard ligand was 0.674 ± 0.84, whereas higher averages were recorded for food-derived peptides, including Seq1 (1.395 ± 1.00), Seq6 (1.557 ± 1.48), and Seq7 (1.543 ± 1.27). The redesigned peptides demonstrated even greater hydrogen bonding capacity, with the cyclic peptide maintaining the highest average (3.058 ± 1.42), followed by the disulfide bridge peptide (1.656 ± 1.57). A closer inspection of the trajectories highlights the dynamic nature of hydrogen bond formation. For the standard ligand, a consistent hydrogen bond network was observed between 75 and 100 ns; however, this gradually diminished toward the later stages of the simulation. Seq1 displayed an initial burst of four hydrogen bonds (0–25 ns), which peaked to five bonds around 100–175 ns, and finally stabilized with two bonds at 200 ns. Seq6 exhibited strong early stability, forming six hydrogen bonds within 0–45 ns, which increased to seven around 125 ns before reducing to two by the end of the simulation. Seq7 maintained relatively stable interactions, with five hydrogen bonds during most of the trajectory, which decreased slightly to three bonds at 200 ns. The cyclic peptide demonstrated superior stability, maintaining a continuous hydrogen bond network ranging between four and eight throughout the entire 200 ns trajectory, and concluding with four bonds at the final frame. The disulfide peptide also showed favorable stability, with hydrogen bond interactions initiating after 25 ns and persisting consistently, ultimately forming six hydrogen bonds by the end of the simulation. Collectively, these results indicate that while the food-derived peptides establish moderate yet consistent hydrogen bonds with TNF-alpha, the designed peptides, particularly the cyclic and disulfide variants, exhibit a more stable and persistent hydrogen-bonding network owing to their structural rigidity and constrained conformations. The findings underscore the therapeutic advantage of cyclic and disulfide-rich peptide designs, which not only stabilize binding but may also improve inhibitory efficacy compared to linear peptides and the standard ligand.

#### 3.7.4 Radius of gyration

The radius of gyration (Rg) was calculated to assess the structural integrity and overall compactness of TNF-alpha in complex with the food-derived and redesigned peptides over a 200 ns simulation (Figure 8C; Table 5). The average Rg values indicated minimal deviations across all systems, reflecting stable folding and compactness of the protein–peptide complexes. The standard ligand maintained an Rg of 1.546 ± 0.006 nm, while the food-derived peptides exhibited values of 1.565 ± 0.01 nm (Seq1), 1.545 ± 0.009 nm (Seq6), and 1.546 ± 0.008 nm (Seq7), suggesting that Seq6 and Seq7 maintained structural compactness comparable to the standard. In contrast, Seq1 showed a slightly higher Rg, indicating minor fluctuations in protein compactness. The redesigned peptides demonstrated the greatest compactness: the cyclic peptide exhibited an Rg of 1.538 ± 0.007 nm, and the disulfide-stabilized analogue showed 1.534 ± 0.01 nm, reflecting enhanced rigidity and tighter packing at the TNF-alpha structure. This increased compactness aligns with observations from RMSD, RMSF, and hydrogen bond analyses, confirming that structural optimization strategies not only stabilize the peptide conformation but also promote a more compact protein–peptide interface.

Collectively, the analysis indicates that both linear and cyclic peptides hold significant therapeutic potential. Among the linear peptides, Seq7 (PPYCTIVPFGIFGTNYR) demonstrated the greatest stability with low RMSD and consistent RMSF, reflecting stable anchoring at key TNF-alpha residues, though minor Rg fluctuations arose from its flexible backbone and subtle protein loop movements. In contrast, the cyclic and disulfide-stabilized peptides exhibited enhanced rigidity, greater compactness, and a stronger, more persistent hydrogen-bond network. While overall Rg differences were modest due to natural loop dynamics, the cyclic designs formed a robust, tightly packed complex, supporting prolonged inhibitory activity within the TNF-alpha binding site.

### 3.8 Metadynamic analysis of the trajectories

#### 3.8.1 Combined PCA–DCCM analysis of protein dynamics

**FIGURE 9:**
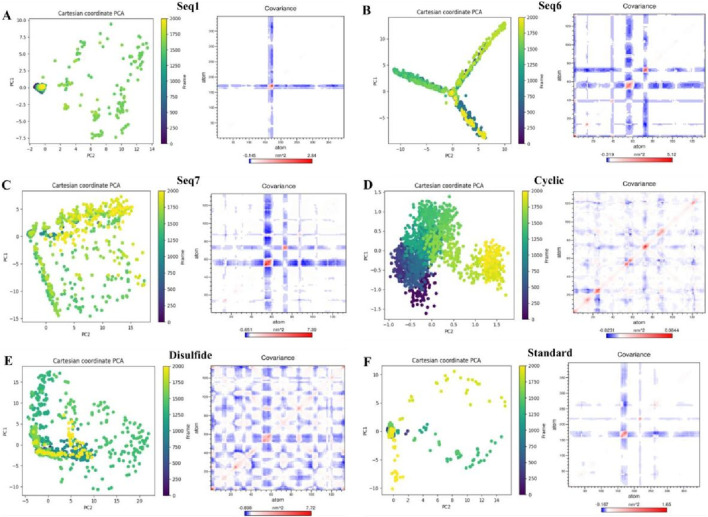
Principal component analysis (PCA) and dynamic cross-correlation matrices (DCCM) of TNF-alpha complexes with food-derived peptides, designed peptides, and the standard inhibitor. For each system **(A)** Seq1, **(B)** Seq6, **(C)** Seq7, **(D)** Cyclic, **(E)** Disulfide, and **(F)** Standard. The left shows PCA plots of the first two principal components (PC1 vs. PC2) colored by simulation frame, illustrating the dominant motions sampled during 200 ns molecular dynamics. The right presents the corresponding DCCM maps depicting correlated (blue) and anti-correlated (red) atomic fluctuations, highlighting differences in collective dynamics across peptide–TNF-alpha complexes.

The dynamically favorable conformational changes of the protein–peptide complexes were explored through principal component analysis (PCA), which captured the dominant motions within the essential subspace. The Cartesian coordinate PCA plots revealed distinct conformational landscapes across the quadrants defined by PC1 and PC2 (Figure 9, left). The PCA plots for food-derived peptides (Seq1, Seq6, and Seq7) revealed a broad distribution spanning multiple quadrants, indicating extensive exploration of the essential subspace. Seq6 and Seq7, in particular, display branched or arc-like patterns that reflect multiple metastable states and pronounced flexibility. Such dispersion denotes an induced-fit binding mode that allows adaptive surface complementarity but incurs a higher entropic penalty and can shorten residence time. In contrast, the cyclic peptide forms a compact cluster confined largely to the first quadrant, demonstrating a pre-organized, low-entropy ensemble, and rigid conformation optimized for stable binding. The disulfide-bridge peptide occupies the first quadrant with a gentle extension into the fourth, signifying intermediate flexibility, more constrained than linear food-derived peptides but less restricted than the cyclic designed.

To evaluate the collective internal motions of the peptide–TNF-alpha complexes, dynamic cross-correlation matrices (DCCMs) were generated, where red regions denote positively correlated residue motions and blue regions indicate anti-correlated motions (Figure 9, right). Regions of strong positive correlation indicate residues that move cooperatively and can form communication pathways, whereas anti-correlated patches reflect hinge-like or compensatory motions that can alter long-range coupling. The standard ligand and Seq1 exhibited only weak, localized correlations, consistent with pocket-filling interactions that preserve the native global dynamics of TNF-alpha. The Seq6 and Seq7 generate red/blue networks across distal regions of TNF-alpha. Signifying allosteric communication and redistribution of conformational fluctuations. Seq1 exhibits mostly weak correlation, consistent with a pocket-filling interaction that leaves the global dynamic network largely intact. The cyclic construct shows localized positive correlations confined to the binding interface, reinforcing its lock-and-key mechanism and localized stabilization of nearby residues. The disulfide variant produces broader correlated/anticorrelated regions than the cyclic peptide, reflecting moderate allosteric effects and controlled global perturbation that may enhance inhibitory efficacy.

The results of our analysis reveal a clear mechanistic distinction between the peptides. Linear food-derived peptides are highly flexible, utilizing an induced-fit mechanism to bind to TNF-alpha. This adaptability, however, leads to broad dynamic changes across the protein, as shown by the DCCM plots. Conversely, the rationally designed cyclic and disulfide peptides progressively limit conformational freedom, enhancing their thermodynamic stability and increasing their residence time. The cyclic peptide achieves this by rigidifying the local binding environment, whereas the disulfide-bridged peptide introduces a balance of rigidity and adaptive allosteric influence. Ultimately, our study demonstrates how targeted structural changes, such as cyclization or disulfide bonds, can directly manipulate a peptide binding and dynamic properties to enhance its inhibitory power.

#### 3.8.2 Free energy landscape profiling

**FIGURE 10:**
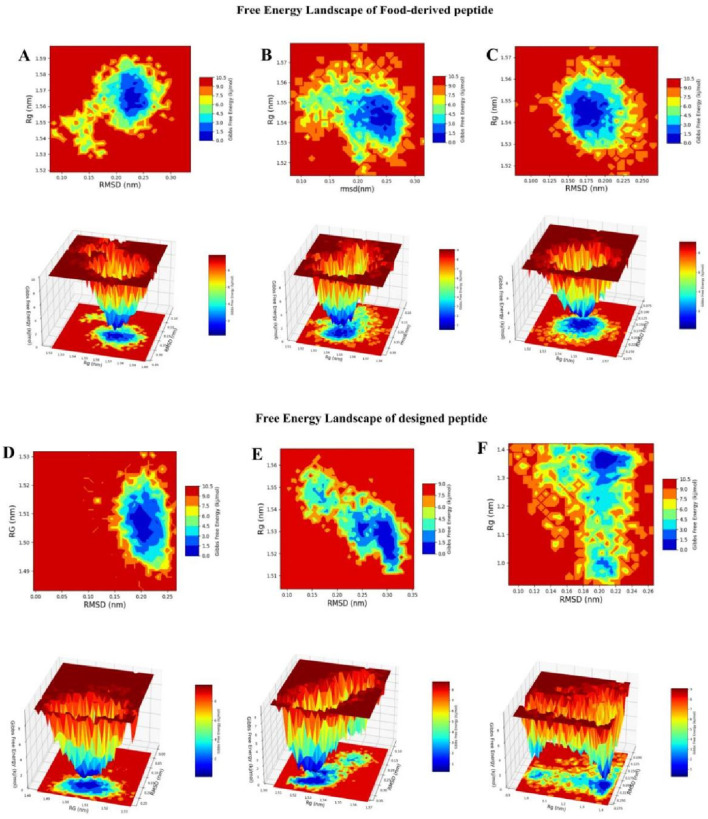
RMSD–Rg-based Gibbs Free Energy Landscape (FEL) plots of peptide–TNF-alpha complexes. The color gradient represents Gibbs free energy (kcal/mol), with blue indicating low-energy, stable conformations and red indicating high-energy, less stable conformations. **(A)** GVDYVRFF (Seq1), **(B)** KIWHHTF (Seq6), **(C)** PPYCTIVPFGIFGTNYR (Seq7), **(D)** cyclic HCLSCSKCRKEM, **(E)** disulfide-stabilized HCLSCSKCRKEM, and **(F)** standard ligand complexed with TNF-alpha.

FEL analysis provided a thermodynamic and mechanistic view of the conformational preferences of linear and cyclic TNF-alpha–peptide complexes. FELs were generated by projecting RMSD and radius of gyration (Rg) values from molecular dynamics trajectories, enabling the identification of energetically favored conformations, folding basins, and structural flexibility. RMSD quantifies structural deviation from a reference state, whereas Rg indicates overall compactness; lower Rg values correspond to more tightly folded, thermodynamically stable states (Figure 10). The reference TNF-alpha–ligand complex displayed a shallow, multi-minima energy surface (RMSD ≈0.18–0.24 nm, Rg ≈ 1.20–1.40 nm, ΔG ≈ 0–2.5 kcal mol^−1^), indicating moderate conformational plasticity while maintaining its trimeric fold. The narrow Rg range suggests that fluctuations arise mainly from local loop motions and inter-subunit adjustments rather than large-scale unfolding Linear, food-derived peptides, exemplified by Seq1 and Seq6, exhibited broad and shallow basins spanning RMSD ≈ 0.20–0.25 nm and Rg ≈ 1.54–1.58 nm with ΔG ≈ 0–2.5 kcal/mol. These features indicate that the peptides sample a wide conformational space with multiple states of comparable energy, reflecting high structural flexibility. Such conformational plasticity is advantageous for dynamic interactions with TNF-alpha, as it allows the peptide to adapt to varying surface geometries and establish induced-fit binding. In contrast, Seq7 displayed a somewhat deeper and more localized basin (RMSD ≈ 0.18–0.22 nm, Rg ≈ 1.53–1.55 nm, ΔG ≈ 0–2.3 kcal/mol), suggesting higher thermodynamic stability and a more defined folding pattern. The cyclic peptide, a rationally designed analogue, exhibited a single, narrow, and deep basin (RMSD ≈ 0.18–0.22 nm, Rg ≈ 1.49–1.52 nm, ΔG ≈ 0–2.0 kcal/mol), reflecting restricted conformational sampling and strong structural pre-organization. Such rigidity favors the maintenance of a specific, high-affinity binding geometry, enhancing the peptide inhibitory potential. The disulfide-stabilized peptide displayed multiple interconnected basins (RMSD ≈ 0.20–0.30 nm, Rg ≈ 1.51–1.53 nm, ΔG ≈ 0–2.5 kcal/mol), representing an intermediate conformational profile that balances moderate flexibility with thermodynamic stabilization.

#### 3.8.3 DSSP analysis of secondary-structure transitions during MD simulations

**FIGURE 11:**
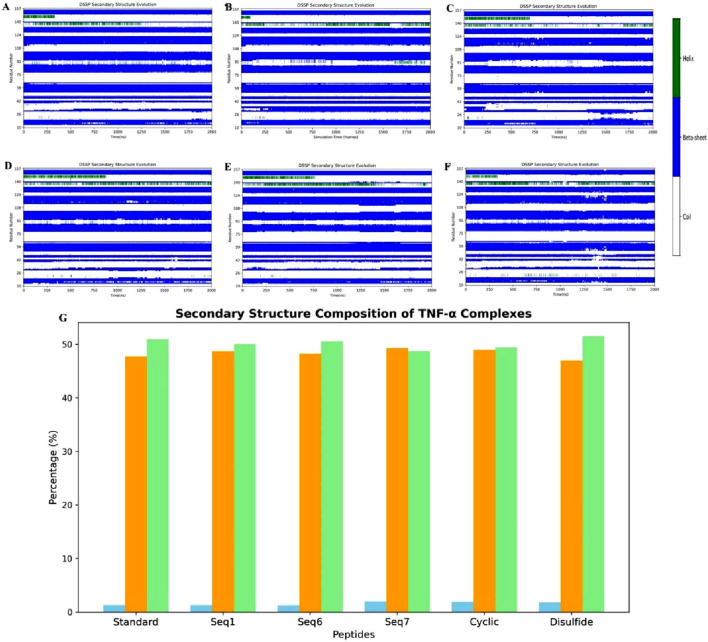
Time-resolved secondary-structure evolution of TNF-alpha–peptide complexes during 200 ns MD simulations. **(A–F)** show DSSP profiles for **(A)** standard complex, **(B)** Seq1, **(C)** Seq6, **(D)** Seq7, **(E)** cyclic peptide, and **(F)** disulfide-bridge peptide, illustrating residue-wise changes in α-helices, β-sheets, and coil regions over time. **(G)** illustrates the quantitative distribution of secondary-structure elements for each system, indicating changes in helical (blue) content while the β-sheet (orange) and coil (light green) framework remains largely conserved.

To further elucidate the peptide binding-induced changes in secondary structure, DSSP-based time evolution profiles were computed using the PyMOL plugin Geo-measure, and the corresponding images were generated with Matplotlib. The results, shown in Figure 11, illustrate the comparative secondary structure dynamics of the standard complex, the food-derived peptide, and the designed analogues. Across all systems, the β-sheet framework within the active site remained largely conserved, confirming that peptide binding does not disrupt the essential scaffold required for TNF-alpha function. In contrast, localized induction of alpha-helical segments was observed in peptide-bound systems, with this effect being most pronounced in Seq7, cyclic, and disulfide-stabilized peptides compared to Seq1, Seq6, and the standard complex. Quantitative analysis of secondary structure composition (Figure 11G) revealed that helicity increased from 1.32% in the standard complex to 2.01% in Seq7, 1.95% in the cyclic peptide, and 1.82% in the disulfide-stabilized peptide, whereas Seq1 (1.31%) and Seq6 (1.28%) remained comparable to the standard. These variations were accompanied by modest adjustments in β-sheet and coil content, but the overall β-sheet dominance was preserved. The increase in alpha-helical content is particularly significant because alpha-helices confer structural rigidity, enhance intramolecular hydrogen bonding, and generate ordered surfaces that promote more specific and stable interactions at the protein–peptide interface. Following protein–peptide binding, helices also play a central role in molecular recognition by presenting a regular array of side chains for complementary surface interactions, thereby stabilizing the binding interface. Such helices are widely recognized as functional motifs in protein–protein interactions, either acting as mimics to competitively disrupt pathological signaling pathways or serving as templates for rational drug design. Overall, these findings demonstrate that while the β-sheet scaffold of TNF-alpha is preserved across all systems, peptide binding particularly by conformationally constrained analogues such as Seq7, cyclic, and disulfide-stabilized peptides induces measurable increases in helical propensity. This structural shift may underpin their enhanced binding affinity, specificity, and overall complex stability, highlighting the therapeutic promise of constrained peptide analogues in modulating TNF-alpha function. Importantly, the food-derived peptide (Seq7) also exhibited a marked increase in alpha-helical content (2.01% helix, 49.28% β-sheet, and 48.71% coil), surpassing the standard and other linear analogues, thereby suggesting that naturally occurring peptides can provide biocompatible scaffolds with favorable conformational properties for therapeutic applications. In parallel, the engineered interacting-residue designed peptides namely the cyclic (1.95% helix, 48.75% β-sheet, 49.40% coil) and disulfide-stabilized analogues (1.82% helix, 46.69% β-sheet, 51.49% coil) displayed the most pronounced helical induction, underscoring how rational design strategies can emulate stabilizing features of native motifs to achieve improved structural resilience and therapeutic potential against TNF-alpha.

The combined metadynamics analyses (PCA, DCCM, FEL, and DSSP) underscore a clear structure–function relationship between peptide topology and TNF-alpha modulation. Linear peptides display high flexibility and broad conformational sampling, inducing widespread dynamic changes across the protein. In contrast, cyclic peptides exhibit restricted motions, occupy well-defined energy minima, and stabilize the local binding environment, with subtle secondary-structure adjustments observed in both cases while preserving the β-sheet core.

### 3.9 Energy determinants of peptide-TNF-alpha interactions: MM-PBSA and residue decomposition

**FIGURE 12:**
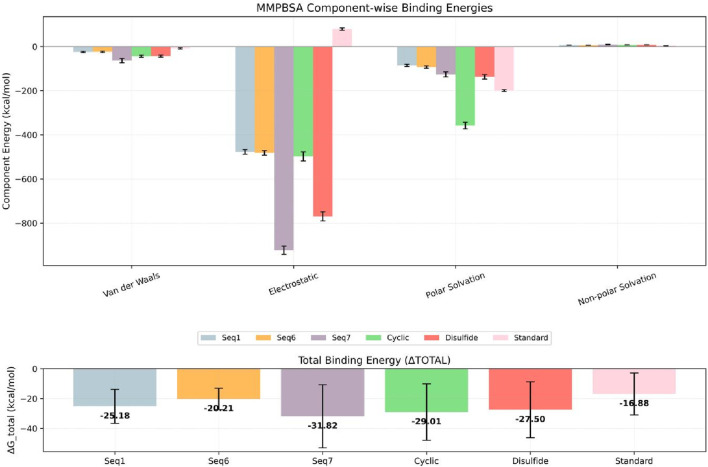
Estimated binding free energies for TNF-alpha protein–peptide complexes calculated using MM/PBSA. The bar plot shows the MM/PBSA-derived van der Waals (ΔEvdW), electrostatic (ΔEelec), polar solvation (ΔEpolar), non-polar solvation (ΔESASA), and total binding free energies (ΔGbind) for TNF-alpha bound to linear peptides Seq1 (blue), Seq6 (yellow), Seq7 (violet), the designed Cyclic peptide (green), the Disulfide-stabilized peptide (brick red), and the standard (pink). Error bars represent the standard deviation across the sampled simulation frames.

A critical determinant of molecular recognition is the binding free energy, which captures the overall thermodynamic favourability of a peptide–receptor complex. This parameter integrates several energetic components such as Van der Waals forces, electrostatic interactions, polar solvation, and non-polar solvation (SASA) to provide a holistic measure of both the strength and stability of binding. In this framework, more negative binding free-energy values signify stronger and more favorable interactions between ligand and target. To probe the binding efficiency and stability of our peptide candidates, we performed MM/PBSA calculations across six TNF-alpha–peptide systems in comparison with a known reference inhibitor (Figure 12; Table 7). The analysis revealed a clear trend in the total binding energies (ΔG_total), following the order: Seq7 (−31.82 kcal/mol^−1^) < Cyclic (−29.01 kcal/mol^−1^) < Disulfide (−27.71 kcal/mol^−1^) < Seq1 (−25.18 kcal/mol^−1^) < Seq6 (−20.21 kcal/mol^−1^) < Standard (−16.88 kcal/mol^−1^). This ranking indicates that the food-derived Seq7 peptide and the rationally designed Cyclic analogue possess the highest binding affinities, exceeding that of the standard inhibitor and even the other food-derived sequences. A deeper decomposition of the energy components highlights the dominant role of van der Waals interactions, which consistently provided the most favorable stabilization across all complexes.

**TABLE 7:** Binding free energies (kcal/mol) of TNF-alpha bound to food-derived and designed peptide and the standard as calculated by MM/PBSA analysis over a 200 ns molecular dynamics simulation.

Energies (kcal/mol)		Food-derived peptide			Designed peptide	
	Standard	Seq1	Seq6	Seq7	Cyclic	Disulfide
Van der Waal Energy (kcal/mol)	−19.82	−33.52	−33.55	−48.76	−40.65	−31.73
Electrostatic energy (kcal/mol)	−136.69	−13.28	−14.12	−14.72	−210.09	−15.11
Polar solvation energy (kcal/mol)	142.28	25.57	31.65	36.82	226.5	28.92
SASA energy (kcal/mol)	−2.65	−3.95	−4.19	−5.17	−4.78	−3.78
Binding energy (kcal/mol)	−16.88	−25.18	−20.21	−31.82	−29.01	−27.71

Notably, Seq7 exhibited the strongest van der Waals contribution (−48.76 kcal/mol^−1^), closely followed by the Cyclic peptide (−40.65 kcal/mol^−1^). Electrostatic interactions, while less prominent in most systems, were strikingly elevated in the Cyclic peptide (−210.09 kcal/mol^−1^) and the Standard inhibitor (−136.69 kcal/mol^−1^), underscoring their critical role in these particular complexes. As anticipated, polar solvation energies were positive, counteracting the binding process and ranging widely from 25.57 kcal/mol^−1^ for Seq1 to a substantial 226.50 kcal/mol^−1^ for the Cyclic peptide. In contrast, the non-polar solvation (SASA) component made a modest but consistent stabilizing contribution of roughly −3 to −5 kcal/mol^−1^ across all systems. Collectively, these results indicate that the most potent peptides achieve a favorable enthalpy–entropy balance by combining extensive hydrophobic packing with selected electrostatic interactions, despite desolvation penalties.

**FIGURE 13:**
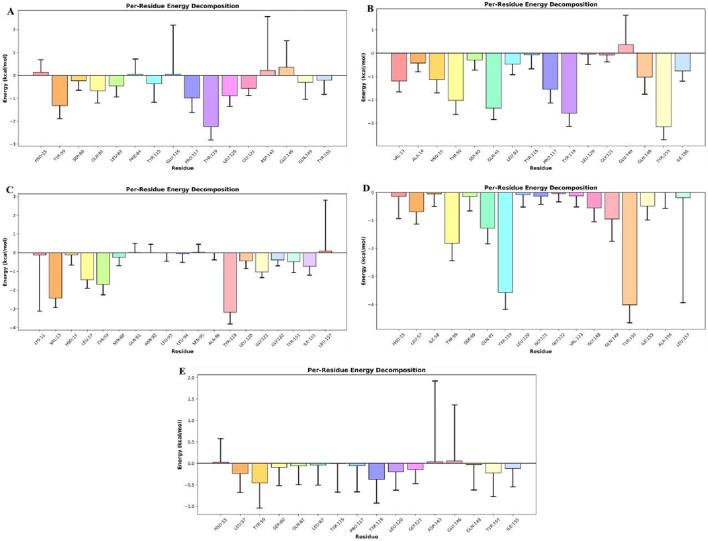
Per-residue contributions to the total binding free energy (ΔG_total) for five peptide–TNF-alpha complexes. Negative values indicate favorable interactions, while positive values indicate unfavorable contributions. show: **(A)** Seq1, **(B)** Seq6, **(C)** Seq7, **(D)** Cyclic peptide, and **(E)** Disulfide-stabilized peptide.

To pinpoint the molecular determinants of this thermodynamic profile, per-residue energy decomposition was performed over the final 200 ns of the MD trajectories. Notably, residues previously reported to interact with TNF-alpha inhibitors contributed substantially to the overall binding free energy (Figure 13). In the Seq1 complex, Tyr59 (−1.31 kcal/mol^−1^) and Tyr119 (−2.24 kcal/mol^−1^) were key contributors. For Seq6, Tyr59 (−2.02 kcal/mol^−1^), Gln61 (−2.35 kcal/mol^−1^), Tyr119 (2.59 kcal/mol^−1^), and Tyr151 (−3.15 kcal/mol^−1^) dominated. The Seq7 complex exhibited strong contributions from Val13 (−2.49 kcal/mol^−1^), Leu57 (−1.44 kcal/mol^−1^), Leu59 (−1.68 kcal/mol^−1^), and Tyr119 (−3.38 kcal/mol^−1^). In the Cyclic complex, Tyr59 (−1.81 kcal/mol^−1^), Gln61 (−1.27 kcal/mol^−1^), Tyr119 (−3.57 kcal/mol^−1^), and Tyr151 (−4.01 kcal/mol^−1^) were dominant contributors, whereas in the Disulfide complex, Tyr59 (−0.45 kcal/mol^−1^) and Tyr119 (−0.38 kcal/mol^−1^) provided modest but notable stabilization. Among these, Tyr119 consistently emerged as the most influential hot-spot, displaying the highest binding contributions in Seq7 and the Cyclic peptide. This per-residue decomposition analysis correlates well with our earlier docking and interacting-residue findings, reinforcing the identification of key hot-spot residues and validating the structural models. The superior thermodynamic stability of Seq7 and the Cyclic analogue can be attributed to their ability to (i) maximize van der Waals packing within the receptor cleft, (ii) exploit key residues such as Tyr119, Gln61, and Tyr151, and (iii) achieve an optimal enthalpic-entropic balance despite polar desolvation costs. Collectively, these results delineate a unified mechanism of TNF-alpha inhibition in which peptide binding is driven by synergistic hydrophobic enclosure and targeted electrostatic interactions anchored by conserved aromatic residues. These mechanistic insights not only validate the computational design strategy but also identify precise structural features, particularly π- π stacking with Tyr119 and complementary interactions with Gln61 and Tyr151, that can guide the rational optimization of next-generation peptide therapeutics targeting TNF-alpha and related inflammatory pathways.

## 4 ADMET evaluation of food-derived and designed peptides

Based on molecular dynamics (MD) simulations and MM/PBSA binding-free energy analysis, Seq7 (linear) and the cyclic peptide were prioritized for detailed ADMET profiling to evaluate their pharmacokinetic behavior and safety (see Table 8). The ADMET analysis revealed comparable profiles for both peptides, with several advantages favoring the cyclic analogue. Both peptides exhibited moderate water solubility (log S = −2.892), poor Caco-2 permeability (−0.434 for linear vs. −0.52 for cyclic), and negligible human intestinal absorption (0%), suggesting limited oral bioavailability. Both were predicted to be P-glycoprotein substrates but not inhibitors, indicating possible active efflux without significant drug–drug interaction risk. The cyclic peptide demonstrated a slightly higher fraction unbound (Fu = 0.398 vs. 0.351) and a greater volume of distribution (log VDss = −0.196 vs. −0.31), reflecting marginally improved tissue distribution. Both peptides showed very low BBB permeability (log BB = −3.189 for linear, −2.892 for cyclic) and CNS permeability (log PS = −7.955 vs. −6.574), indicating minimal CNS exposure. Metabolic predictions indicated that the linear peptide is a CYP3A4 substrate whereas the cyclic peptide is not, suggesting improved metabolic stability for the cyclic form. Excretion analysis showed that the cyclic peptide had lower total clearance (−1.823 vs. −0.381 log mL/min/kg) and a longer half-life (0.88 vs. 0.653), indicating slower elimination and prolonged systemic exposure. Toxicity predictions showed both peptides to be non-mutagenic (negative AMES test), non-carcinogenic, and with similar oral acute toxicity (LD_50_ = 2.482 mol/kg) and maximum tolerated dose (∼0.44 log mg/kg/day). However, the linear peptide was predicted to be a hERG II inhibitor, whereas the cyclic peptide showed no hERG inhibition, suggesting a lower cardiotoxicity risk. Collectively, these findings indicate that the cyclic peptide has a more favorable ADMET profile, with improved metabolic stability, longer systemic retention, and enhanced safety characteristics.

**TABLE 8:** Predicted ADMET properties of the selected peptide from the pkCSM server.

Properties		Peptide sequences	
		Linear (Seq7)	Cyclic
Absorption	Water solubility (log mol/L)	−2.892	−2.892
	Caco2 permeability ((log Papp in 10–6 cm/s)	−0.434	−0.52
	Intestinal absorption (human) (%)	0	0
	P-glycoprotein substrate	Yes	Yes
	P-glycoprotein I inhibitor	No	No
	P-glycoprotein II inhibitor	No	No
Distribution	Fraction unbound (human) Numeric (Fu)	0.351	0.398
	VDss (human) (log L/kg)	−0.31	−0.196
	BBB permeability (log BB)	−3.189	−2.892
	CNS permeability (log PS)	−7.955	−6.574
Metabolism	CYP3A4 substrate/inhibitor	Yes/No	No/No
	CYP1A2 inhibitor	No	No
	CYP2D6 substrate/inhibitor	No/No	No/No
	CYP2C9 inhibitor	No	No
Excretion	Total Clearance (log mL/min/kg)	−0.381	−1.823
	Half-life estimation (t^1/2^)	0.653	0.88
Toxicity	AMES toxicity	No	No
	Max. tolerated dose (human) (log mg/kg/day)	0.438	0.439
	hERG I/II inhibitor	No/Yes	No/No
	Oral Rat Acute Toxicity (LD50) (mol/kg)	2.482	2.482
	Oral Rat Chronic Toxicity (LOAEL) (log mg/kg_bw/day)	9.662	9.357
	Carcinogenicity	No	No
	T.Pyriformis toxicity (log ug/L)	0.285	0.285

## 5 Mechanistic insights into linear and cyclic peptide architectures for TNF-alpha inhibition

The structural topology of a peptide is a critical determinant of its binding strength, conformational stability, pharmacokinetic behavior, and overall inhibitory efficiency against TNF-alpha. For a therapeutic peptide to be effective, it must not only establish high-affinity interactions with TNF-alpha but also maintain sufficient stability in systemic circulation to ensure prolonged biological activity. To address this, both linear (Seq7) and rationally designed cyclic peptides were systematically evaluated using molecular dynamics (MD) simulations, free energy landscape (FEL) analysis, and ADMET predictions, enabling a comprehensive comparison of their structural, thermodynamic, and pharmacokinetic attributes.

Seq7 exhibited substantial conformational adaptability during MD simulations, engaging multiple hotspot residues within the TNF-alpha binding groove. Despite this flexibility, it maintained low RMSD and consistent RMSF values, indicating a relatively stable backbone. Its flexibility allowed transient hydrogen-bond formation and residue-level adjustments that supported binding, but this same adaptability resulted in higher radius of gyration (Rg) values, suggesting a less compact complex. Principal Component Analysis (PCA) and FEL mapping revealed that Seq7 explored a broader conformational space and occupied shallower free-energy basins, consistent with a dynamic but entropically more costly binding mode. While such flexibility may facilitate accommodation of receptor plasticity and point mutations, it can also lead to faster dissociation and lower kinetic stability.

Conversely, the cyclic peptide was rationally engineered by incorporating key hotspot residues from the TNF-alpha–TNFR1 interface (HCLSCSKCRKEM), effectively mimicking the natural receptor epitope. This interface-guided design pre-organizes the peptide into a geometry that closely aligns with the TNF-alpha binding surface, thereby reducing internal degrees of freedom and minimizing entropic penalties during complex formation. MD simulations revealed consistently lower Rg values for the cyclic peptide–TNF-alpha complex, reflecting improved structural compactness, while FEL profiling showed a deeper, well-defined minimum indicative of a thermodynamically more favorable and kinetically stable binding state. Furthermore, H-bond analysis and MM-PBSA calculations demonstrated that the cyclic peptide formed more persistent hydrogen-bond networks and exhibited stronger van der Waals and electrostatic interactions, supporting the notion of a more robust and long-lived complex. These observations are consistent with the findings of (Li et al., 2021), who reported that cyclic RGDfV–integrin αvβ3 complexes possess higher rupture forces and slower dissociation rates compared to their linear counterparts. This correlation supports the mechanistic basis of our results, where cyclization enhances conformational rigidity, reduces entropic cost, and stabilizes peptide–protein interactions, ultimately conferring superior kinetic stability and prolonged inhibitory persistence in the TNF-alpha system.

**FIGURE 14:**
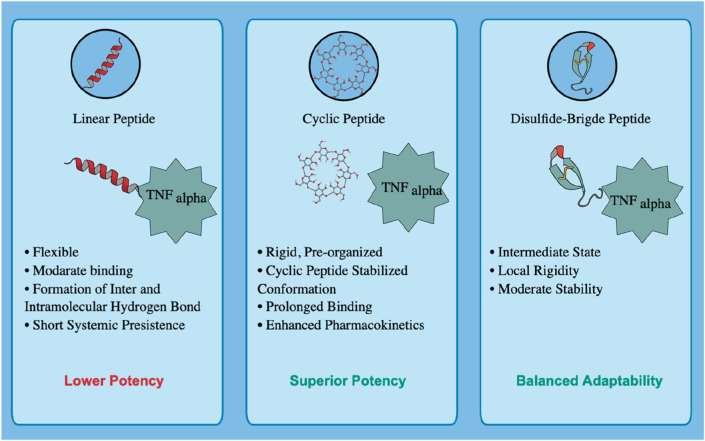
Comparative schematic of peptide architectures evaluated for TNF-alpha inhibition based on our results.

These structural insights were strongly corroborated by ADMET predictions. The cyclic peptide exhibited superior pharmacokinetic properties, including improved plasma stability (t^1^/^2^ = 0.88 vs. 0.65 for Seq7), reduced systemic clearance (log CL = −1.82 vs. −0.38), and slightly enhanced blood–brain barrier penetration (logBB = −2.89 vs. −3.18) and CNS permeability, suggesting prolonged systematic circulation and improved tissue distribution. Similar findings by (Alaofi et al., 2016; Bogdanowich-Knipp et al., 1999) further validate the observed enrichment in the half-life and conferred resistance to proteolytic degradation for the cyclic peptide in our study. Whereas the disulfide-bridge peptides occupy an intermediate state, combining localized rigidity from the disulfide bond with global flexibility elsewhere. They form fewer persistent hydrogen bonds than cyclic peptides and sample broader conformational space, resulting in intermediate free-energy minima. This balance of rigidity and adaptability produces moderate binding stability and pharmacokinetic properties, distinguishing them from fully cyclic or linear scaffolds. Our results corroborate the principle, highlighted by studies on NGR-containing peptides (Negussie et al., 2010), that a peptide biological activity is determined by the conformational stability of its bioactive motif. Our study shows that while the linear Seq7 displayed adaptability, it had lower kinetic stability. In contrast, the cyclic peptide achieved pre-organization and persistent binding. These results provide a coherent mechanistic understanding. Linear peptides, though flexible, suffer from higher entropic penalties and shorter systemic persistence. Cyclic peptides, due to their rigid architecture, achieve deeper energy minima, resist proteolytic degradation, and display superior pharmacokinetic performance. Disulfide-bridge peptides, in turn, occupy an intermediate state. Compared with clinically approved biologics such as Infliximab, the peptide scaffolds identified in this study offer several advantages. Although Infliximab is an FDA-approved TNF-alpha inhibitor with proven clinical utility, it exhibits less than 50% sustained efficacy in certain patient populations and has been associated with increased risk of congestive heart failure, severe infusion reactions, and immune-mediated adverse effects. Moreover, as a large, protein-based biologic, Infliximab requires intravenous administration and faces challenges related to delivery, stability, and immunogenicity (Sahu et al., 2024). In contrast, the food-derived and rationally designed peptide scaffolds developed here demonstrate predicted advantages in bioavailability, systemic stability, and reduced immunogenic potential, owing to their small molecular size and optimized cyclic architecture. This combination of favorable attributes underscores that naturally occurring linear bioactive peptides can be cyclized to stabilize their active conformations, enhance binding affinity, prolong residence time at the TNF-alpha interface, and leverage the pharmacokinetic advantages inherent to cyclic architectures. Collectively, these findings suggest that rationally designed cyclic peptides represent a safer, more versatile, and potentially oral alternative to injectable protein-based inhibitors such as Infliximab. This approach could transform food-derived peptides into potent, long-lasting therapeutic scaffolds for TNF-alpha inhibition and, by extension, provide a promising strategy to combat chronic inflammation underlying cardiovascular disease Figure 14.

## 6 Future directions, limitations, and opportunities in peptide therapeutics

The pharmacological relevance of the food-derived peptide is supported by its known anti-inflammatory and antioxidant activities; however, its direct inhibition of TNF-alpha and the efficacy of the designed cyclic analog require experimental validation. Both peptides should be synthesized and evaluated using ITC or MST to determine TNF-alpha binding affinity. Functional assessment can be performed through ELISA-based cytokine inhibition assays in LPS-stimulated macrophages, followed by *in vivo* validation in ApoE^−/−^ or LDLR^−/−^ mouse models to assess bioavailability, stability, and cardioprotective efficacy. These studies will serve as essential validation steps to translate computational predictions into therapeutic applications. Despite their clear pharmacological advantages, including enhanced conformational rigidity, proteolytic stability, and target-binding affinity. The cyclic and disulfide-stabilized peptides pose synthetic challenges. These include low synthetic yields, complex purification processes, high cost, and sequence-dependent folding constraints, particularly in peptides containing multiple cysteine residues. However, advances in chemoselective ligation, enzymatic cyclization, and orthogonal protection strategies are progressively mitigating these limitations, improving yield, reproducibility, and scalability for the synthesis of bioactive cyclic peptide therapeutics (Isidro-Llobet et al., 2019; Zhang et al., 2019).

From a computational perspective, AI-based peptide screening and generative modeling have emerged as transformative tools for drug discovery, capable of exploring vast sequence spaces and accelerating lead optimization (Goles et al., 2024). Nevertheless, the predictive accuracy of these models critically depends on the quality, diversity, and representativeness of their training data (Hanna et al., 2025; Zhai et al., 2025). Currently, no curated database specifically catalogues food-derived peptide–TNF-alpha interactions, which constrains the accuracy, generalizability, and validation of computational models. Establishing such databases annotated with experimentally verified activity, structural, and binding data would significantly improve AI training, model benchmarking, and predictive performance in the development of inflammation-targeted peptide therapeutics. Future research should focus on integrating AI-driven prediction with physics-based molecular simulations to provides a complementary strategy that merges computational scalability with detailed mechanistic and energetic understanding. Incorporating experimentally validated TNF-alpha interaction models into emerging generative deep docking frameworks (Zhao et al., 2025), will further refine predictive precision and expand the applicability of virtual screening pipelines. In parallel, establishing standardized benchmarks, open-access repositories, and collaborative research networks will be essential to ensure reproducibility, data transparency, and effective knowledge sharing across the peptide research community. Recent advancements in computational methodologies and deep learning frameworks (Wang et al., 2025; Zhao et al., 2024; 2022) underscore the growing potential of these integrated approaches to rationally design next-generation peptide therapeutics. Collectively, these technological and methodological innovations promise to accelerate the discovery of scalable, structurally stable, and mechanistically validated TNF-alpha inhibitors, thereby offering a transformative path toward safe and effective treatments for chronic inflammatory diseases.

## 7 Conclusion

The inherent advantages of peptides, including their ease of synthesis, low toxicity, and high target specificity, motivated this study to explore peptide-based inhibition of TNF-alpha. We successfully identified a food-derived bioactive peptide and rationally designed a cyclic analog from the TNF-alpha–TNFR1 interface, both of which proved to be potent inhibitors capable of blocking downstream TNF-alpha signaling. By preventing the TNF-alpha/TNFR1 interaction, these peptides are expected to attenuate pro-inflammatory cascades without triggering downstream signaling events such as NF-κB activation, thereby reducing the risk of secondary inflammatory amplification. This work demonstrates a powerful strategy combining the intrinsic bioactivity of naturally occurring peptides with interface-guided cyclization to create effective therapeutic candidates. Our findings align with existing literature, which consistently reports that cyclic peptides possess superior stability and active-site retention compared to their linear counterparts. Computational analyses revealed that the designed interface cyclic peptide achieved deeper free-energy minima, prolonged retention within the binding pocket, and enhanced hydrogen-bond persistence. In contrast, the food-derived linear peptide, while maintaining strong binding, displayed greater conformational flexibility. This suggests that cyclization can further improve the structural stability and pharmacokinetic performance of naturally active food-derived peptides. Because the identified food-derived peptide already has reported bioactivities, its cyclization could potentially enhance these anti-inflammatory and anti-proliferative effects. This study provides a foundational framework for both discovering diverse food-based peptide inhibitors and designing potent, interface-derived cyclic peptides as TNF-alpha antagonists. As a next step, experimental validation of the designed cyclic peptide is warranted to confirm its real-time clinical applicability and to fully assess its therapeutic potential. These findings also resonate with the United Nations Sustainable Development Goals, particularly SDG 3 (Good Health and Wellbeing) and SDG 9 (Industry, Innovation, and Infrastructure), by advancing sustainable, innovation-driven strategies for cardiovascular drug discovery.

## Data Availability

The raw data supporting the conclusions of this article will be made available by the authors, without undue reservation.
