# Supplementary material for: Food-derived linear vs. rationally designed cyclic peptides as potent TNF-alpha inhibitors: an integrative computational study
Source: Front Bioinform. 2025 Nov 18;5:1716375. doi: 10.3389/fbinf.2025.1716375 (PMC12669231; doi:10.3389/fbinf.2025.1716375)
Supplement: Supplementary file 1 [file Supplementaryfile1.docx]

**Food-Derived Linear vs. Rationally Designed Cyclic Peptides as Potent TNF-alpha Inhibitors: A Integrative Computational Study**

Manisha Shah^1^, Sivakumar Arumugam^1^*

*Correspondence: [siva_kumar.a@vit.ac.in](mailto:siva_kumar.a@vit.ac.in)

1. Department of Bio Sciences, School of Bio Sciences and Technology, Vellore Institute of Technology Vellore, Tamil Nadu, 632014, India.

1. **Materials and Methods**

**1.1 Three-dimensional structural prediction of food-derived peptide**

Three-dimensional structures of the selected peptides were predicted using the PEP-FOLD4.0 web server (Rey et al., 2023). PEP-FOLD4 employs a de novo modeling approach based on the sOPEP coarse-grained force field to generate energetically favorable peptide conformations from amino acid sequences. In this workflow, peptides are represented as overlapping four-residue fragments that serve as local structural prototypes. Sequence profiles generated via PSI-BLAST are converted into structural alphabet (SA) profiles using a support vector machine classifier, with each SA state corresponding to a local structural motif. Conformational space is sampled using Hidden Markov Model-based algorithms, and trajectories of individual fragments are assembled iteratively to construct complete peptide models. These structures are refined using Monte Carlo simulations, in which fragment swaps optimize geometry, while a greedy algorithm selects low-energy conformations guided by the sOPEP force field. For this study, default PEP-FOLD4 settings were applied, with 200 conformations generated per peptide, and the top five lowest-energy structures were retained for downstream docking and molecular dynamics analyses. Ionic strength and pH effects were incorporated through the Debye–Hückel formalism. This workflow ensures accurate structural modeling and reproducible prediction of bioactive peptide conformations.

**Table 1**: Physicochemical filter criteria and justification for food-derived TNF-alpha inhibitory peptides.

| Filter Criterion | Applied Threshold | Justification (TNF-alpha context) |
| --- | --- | --- |
| Isoelectric point (pI) | 5.0 – 10.0 | Maintains peptides in a physiologically compatible ionization state (pH ~7.4). This range supports sufficient charge flexibility to adapt to the predominantly polar/neutral environment of the TNF-alpha binding interface while preserving solubility. |
| GRAVY (hydropathicity) | −0.6 to +0.6 | Selects for amphipathic balance, which is critical for mediating stable protein–protein contacts with TNF-alpha. Excessively hydrophobic peptides risk aggregation, while overly hydrophilic peptides may fail to anchor effectively at the binding groove. |
| Molecular Weight (MW) | 450 – 3500 Da | Ensures peptides are small enough to access the shallow binding grooves of TNF-alpha while avoiding very small fragments that may lack structural robustness. Larger peptides (>3.5 kDa) are more prone to instability and reduced bioavailability. |
| Charge balance | Neutral to moderately positive | Promotes favorable electrostatic complementarity with TNF-alpha polar binding residues. A slight positive skew enhances peptide–protein attraction without introducing excessive charge that may destabilize solubility or structure. |
| Instability index | Stable only (index < 40) | Filters for peptides predicted to remain stable under physiological conditions, thereby increasing the likelihood that food-derived candidates maintain their conformation and inhibitory activity against TNF-alpha. |

**Table 2:** Different biological properties of the interacting residues

| Peptide Sequence | Bioactivity score | Anti-inflammatory prediction | Cell penetrating | Mutation |
| --- | --- | --- | --- | --- |
| HCLSCSKCRKEM | 0.762 | AIP | Non-CPP | No Mutation |
| TYLYN | 0.204 | AIP | Non-CPP | No Mutation |
| DCRECESG | 0.202 | AIP | Non-CPP | No Mutation |
